# Reference Frames and 3-D Shape Perception of Pictured Objects: On Verticality and Viewpoint-From-Above

**DOI:** 10.1177/2041669516637286

**Published:** 2016-06-29

**Authors:** Els V. K. Cornelis, Andrea J. van Doorn, Johan Wagemans

**Affiliations:** Department of Brain & Cognition, Laboratory of Experimental Psychology, University of Leuven (KU Leuven), Leuven, Belgium; Psychologische Functieleer, Faculteit Sociale Wetenschappen, Universiteit Utrecht, Utrecht, The Netherlands; Department of Brain & Cognition, Laboratory of Experimental Psychology, University of Leuven (KU Leuven), Leuven, Belgium

**Keywords:** verticality, viewpoint-from-above prior, pictorial relief, picture orientation, observer orientation, environmental reference frame, viewer-centered reference frame, pictorial perception, 3-D shape perception

## Abstract

Research on the influence of reference frames has generally focused on visual phenomena such as the oblique effect, the subjective visual vertical, the perceptual upright, and ambiguous figures. Another line of research concerns mental rotation studies in which participants had to discriminate between familiar or previously seen 2-D figures or pictures of 3-D objects and their rotated versions. In the present study, we disentangled the influence of the environmental and the viewer-centered reference frame, as classically done, by comparing the performances obtained in various picture and participant orientations. However, this time, the performance is the pictorial relief: the probed 3-D shape percept of the depicted object reconstructed from the local attitude settings of the participant. Comparisons between the pictorial reliefs based on different picture and participant orientations led to two major findings. First, in general, the pictorial reliefs were highly similar if the orientation of the depicted object was vertical with regard to the environmental or the viewer-centered reference frame. Second, a viewpoint-from-above interpretation could almost completely account for the shears occurring between the pictorial reliefs. More specifically, the shears could largely be considered as combinations of slants generated from the viewpoint-from-above, which was determined by the environmental as well as by the viewer-centered reference frame.

## Introduction

### The Effect of Orientation on the Shape Percept

A well-known and striking example illustrating the importance of the orientation of an object on its appearance is a square turning into a diamond when being oriented onto one of its corners (Mach, 1886/1959). The object, a figure with four equal sides and equal corners, drastically changes in appearance, although nothing but its orientation has altered. Apparently, the appearance of the object changes to such an extent, that another name has been assigned to the essentially identical figure. When having changed the orientation of an object, the orientation has been altered *in relation to* a reference. In this sense, the orientation of an object is a relative concept, and changes with regard to the viewer (i.e., retina, head, or body) as well as the environment (including visual landmarks or cues, such as walls, ceiling, and the floor of the room, but also gravity on earth).

In most everyday conditions, the viewer-centered reference frame coincides with the environmental reference frame. In studies in which the environmental reference frame and the viewer-centered reference frame were disentangled, the superiority of one of both reference frames, the environmental reference frame, or the viewer-centered reference frame was found to depend, for instance, on the complexity of the stimuli (Rock & Heimer, 1957), the task (McMullen & Jolicoeur, 1990), the instructions (Attneave & Reid, 1968; Rock & Heimer, 1957), the surround (such as a rectangular or a circular surround: Corballis, Anuza, & Blake, 1978), the absence of the gravitational reference frame (Friederici & Levelt, 1990; Leone, Lipshits, McIntyre, & Gurfunkel, 1995; Rock & Heimer, 1957), and the conditions of attention (Rock & Nijhawan, 1989). In addition, young infants seem to use the viewer-centered reference frame more than the environmental reference frame (Jouen, 1985; Kushiro, Taga, & Watanabe, 2007). Low-level visual phenomena such as the (visual) tilt after effect (Knapen, Rolfs, Wexler, & Cavanagh, 2010; Mathôt and Theeuwes, 2013; Rieser & Banks, 1981), the visual acuity (Banks & Stolarz, 1975), and the visual performance fields (Corbett & Carrasco, 2011) seem to be largely dependent on the retinal image, and thus the viewer-centered reference frame. Moreover, the detection of symmetry in dot patterns (Corballis & Roldan, 1975; Fisher & Bornstein, 1982) or in random dot stereograms (Julesz, 1971) and the detection of repetition (Corballis, Zbodroff, & Roldan, 1976) were found to be generally more relying on the viewer-centered reference frame (even retinal, according to most of these authors). Wenderoth and Hickey (1993), Howard, Bergström, and Ohmi (1990), and Mamassian, Jentzsch, Bacon and Schweinberger (2003) stated that the perception of shape from shading (and the light-from-above prior) was largely related to a head centered or retinotopic reference frame. However, if the task included an explicit shape judgement of shape-from-shading stimuli, a partial compensation for head tilt relative to gravity occurred, which is in contrast to, for instance, visual search tasks using the same stimuli (Adams, 2008). In addition, participants rotating a single shaded disk until it appeared maximally convex in different conditions of presentation (i.e., with the participant in upright position or on the side, in either a normal or a tilted room) were influenced by gravitational, visual, and bodily cues (Jenkin, Jenkin, Dyde, & Harris, 2004). Troje (2003) asserted that, besides the low-level or more implicit processes as mentioned earlier, the tasks that involve more sophisticated form discrimination, such as face perception and biological motion, are highly influenced by the viewer-(i.e., body) centered reference frame. Aside from the studies on face perception and on biological motion, naming familiar objects was reported to be more dependent on the viewer-centered reference frame than on the environmental reference frame (McMullen & Jolicoeur, 1990). The “perceptual upright” (as related to the upright orientation of objects) was largely influenced by the viewer-centered reference frame, more specifically by the body orientation, and to a lesser (although still considerable) extent by the visual information from the scene as well as the gravitational information (Dyde, Jenkin, & Harris, 2006).

In contrast with the “perceptual upright”, tasks comprising some kind of detection of line orientation would make more use of the environmental reference system (Troje, 2003). Superiority of the environmental reference system was supported in studies on the Goldmeier effect (Attneave & Olson, 1967; Ferrante, Gerbino, & Rock, 1995; Rock & Leaman, 1963), on the visual vertical (Dyde et al., 2006) and horizontal (Asch & Witkin, 1948), and on the matching of surface orientations (Sedgwick & Levy, 1985). Further studies in which the environmental reference frame was observed to be predominant are, for instance, discrimination studies (between alphanumerical or nonsense letter-like figures and their mirror-reflected counterparts: Corballis, Nagourney, Shetzer, and Stefanatos (1978); between left-facing and right-facing familiar objects and between alphanumerical characters and their mirror-reflected counterparts: McMullen and Jolicoeur (1990); between distractor views of a tube-like, 3-D object and target views of the same object, rotated in depth: Waszak, Drewing, and Mausfeld (2005), and recognition studies (of previously learned nonsense figures in a specific orientation: Rock, 1956; Rock & Heimer, 1957). Furthermore, Hinton and Parsons (1988) observed that in order to discriminate between reflected and identical 3-D objects, participants rotated the comparison object mostly according to the environmental reference frame (i.e., the visual information in the room), for both the mental as well as the physical rotation task.

In the studies mentioned earlier, one was generally able to deduce the magnitude of the orientation effects from the recognition and detection performances, but not the nature of the effects. The nature of the differences between the visual percepts obtained from stimuli shown in various (stimulus or participant) orientations could not be derived. In the present study, we used a different approach to study the importance of the environmental and the viewer-centered reference frame in visual perception. We externalized the 3-D shape percept of the depicted object by using a gauge figure task. The externalization of the percept enabled us to examine not only the extent of the orientation effects but also the nature of those effects.

Previously, we used the gauge figure method to study plane orientation effects on the shape percept of the depicted object (Cornelis, van Doorn, & Wagemans, 2009). Externalized 3-D percepts of the depicted object, so-called *pictorial reliefs*, were gathered for pictures presented in different orientations. Altering the picture orientation evidently means that the orientation of the picture has not only been changed with regard to the environmental but also with regard to the viewer-centered reference frame (provided that the participant orientation remains constant). Consequently, both reference frames could have contributed to the differences found between the pictorial reliefs based on the pictures shown in different orientations.

To answer the questions that were raised by the previous study, we investigated the respective contributions of the environmental and the viewer-centered reference frame using the previously used stimuli (Cornelis, van Doorn, & de Ridder, 2003; Cornelis et al., 2009). To this end, we not only varied the orientation of the picture but also varied the orientation of the participant. A large number of studies have focused on the role of the reference frames on visual perception and object recognition (see earlier). However, we do not know of any study focusing on the importance of the reference frames in 3-D shape perception of real (depicted) objects. Although one may expect a contribution of both reference frames on the basis of previous studies (e.g., Adams, 2008; Jenkin et al., 2004), substantial methodological differences with these earlier studies make it difficult to formulate clear-cut hypotheses about the outcome of the present study. To allow for unexpected but interesting effects to emerge from this large-scale study, an extensive set of comparisons between all the relevant conditions was carried out.

### Present Study

In the present study, we dissociated the viewer-centered reference frame from the environmental reference frame by varying the orientation of the participant. The variation of the orientation of the participant consisted of sitting straight up, lying on the right-hand side, and lying on the left-hand side.^1^ In addition, the pictures were presented in four different orientations; the original photograph was rotated by 0°, 90°, 180°, or 270°. Pictorial reliefs were gathered for all possible combinations of picture orientations and participant orientations.

To unravel the influence of the environmental and the viewer-centered reference frame, we compared every two pictorial reliefs, obtained from different conditions, and thus differing with respect to the picture orientation (Rot0, Rot90, Rot180, Rot270) and the participant orientation (Rot0, Rot90, Rot180, Rot270). Since we scrutinized all possible comparisons between the pictorial reliefs by means of general statistical analyses as well as more detailed analyses, this yields a huge number of comparisons, plots, and discussions. To help the reader to maintain an overview, we have produced several explanatory figures to make it easier to understand the variables and conditions (and return to them when needed), and we have introduced some additional layout features: Table 1 in the Supplement contains an overview of notations and concepts; in the labels, symbols, and graphs, we used consistent color coding; additionally, in the text, technical sections are indicated by gray boxes, conclusions by yellow boxes.

**Table 1.**
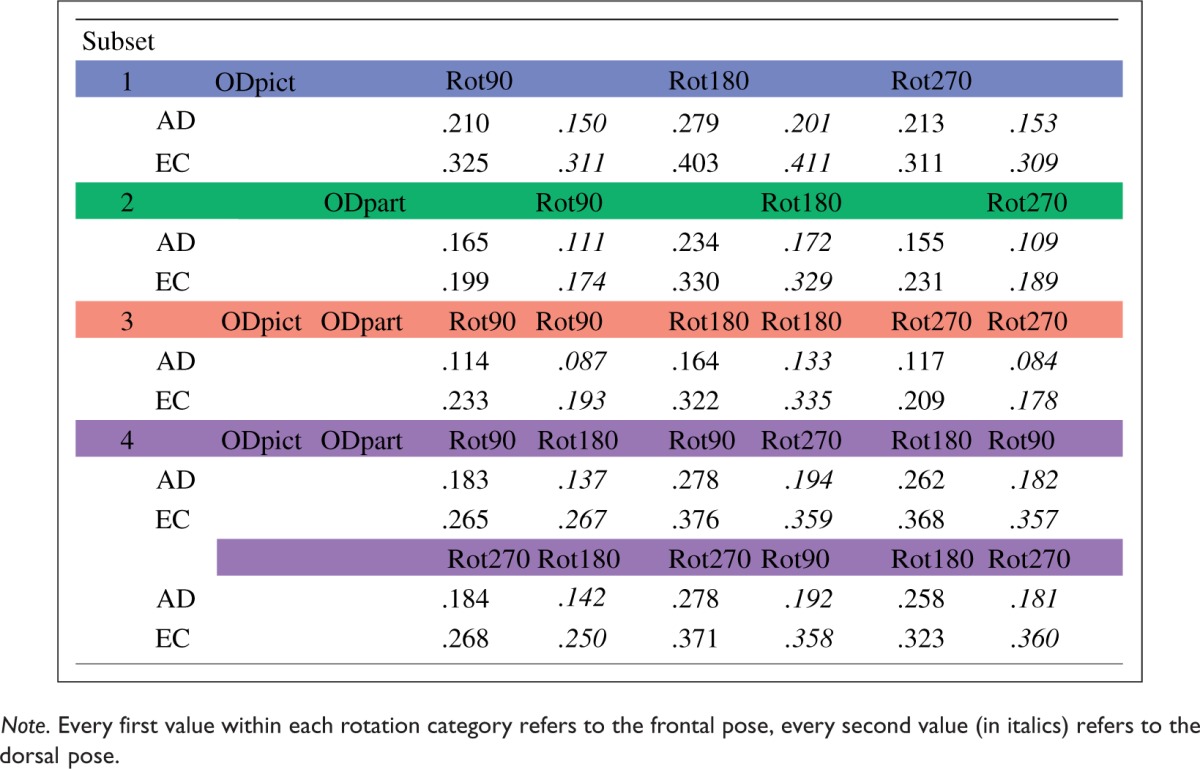
Mean Magnitudes of the Shears.

## Experiment

### Stimuli

We used photographs of a female shop-window mannequin. As was already mentioned in the predecessors of the present study (Cornelis et al., 2003, 2009), we chose to depict this specific object because of its generic geometrical structure and smooth surface, with a good balance between smooth and sudden curvature changes on the surface as well as gradual and steep surface orientation changes relative to the camera. The mannequin showed diffuse reflection and was lit from left above. The photographs were taken in gray-scale with a Nikon Coolpix 990 digital camera.

One of the two photographs pictured a frontal view of the torso (the 60° pose or Pose 60); the other photograph pictured a dorsal view (the 280° pose or Pose 280). Pose 60 and Pose 280 differed by 140° around the vertical axis. Position of the torso, camera and light source remained constant between the shots of the two views of the torso: The difference in pose was the only variation between photographs. The two photographs of the torso were previously used in Cornelis et al. (2003, 2009).

### Participants

The two participants (A.D. and E.C., both authors) had normal or corrected-to-normal vision. Note that this is the kind of study one can hardly ask naïve participants to execute: Each participant performed the gauge setting task during 36 hours. In a laborious and time-consuming paradigm like the one we used here, one can wonder how reliable and valid the data obtained with naïve observers (who usually do this for payment) would be. It requires tremendous effort and sustained attention to be willing to go through a long series of trials with basically the same task and stimulus, except for orientation and position changes. In addition, with a direct visual task such as the gauge figure task, it is infeasible to visualize the “right” answer on basis of some conscious, mental, overall image of the torso depicted. Therefore, we think we are all (visually) “naïve” when performing a direct visual task such as the gauge figure task. We consider it nearly impossible that some kind of pre-knowledge about possible hypotheses and expectations would have influenced the results.

### Procedure

#### Experimental Manipulations

##### Variation in participant orientation

One of the two experimental manipulations involved the orientation of the participant. The various orientations of the participant consisted of the 0° orientation (VF0), the 90° orientation (VF90) and the 270° orientation (VF270; with V for viewer; F for fiducial).

The VF0 orientation was the upright position of the participant sitting in a chair with the chin put in a chin rest. The VF90 and VF270 orientations were obtained by putting the participant on a bed, on the right-hand side and the left-hand side, respectively. We carefully arranged that in all participant orientations, the center of the picture was straight ahead with respect to the viewpoint of the participant. The eye-to-screen distance was kept constant at 50 cm.

##### Variation in Picture Orientation

The second experimental manipulation concerned the orientation of the picture. The various orientations of the picture were obtained by rotating the photograph by 0 (F0), 90 (F90), 180 (F180), and 270° (F270; with F for fiducial).

**Figure 1.**
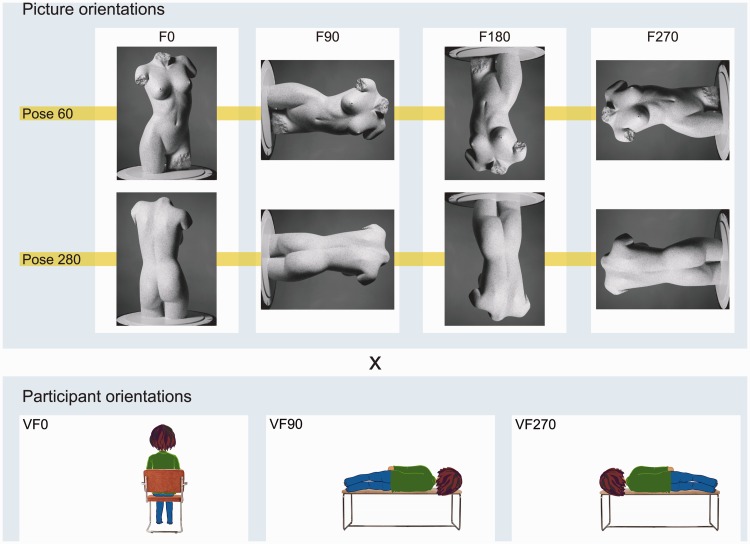
The combinations of picture orientations and participant orientations used in the experiment. For each pose, there were 12 possible combinations of picture and participant orientations. Top row, the picture orientations: the original photographs (F0) of the frontal view (Pose 60), and the dorsal view (Pose 280) of the same shop window mannequin, and their rotated versions (F90, F180, and F270). Bottom row, the participant orientations: sitting straight up (VF0), lying on the right-hand side (VF90), and lying on the left-hand side (VF270).V, viewer; F, fiducial; 0, not rotated; 90, rotated by 90°; 180, rotated by 180°; 270, rotated by 270°.

Data were gathered for all possible combinations of picture orientations and participant orientations (Figure 1).

### Gauge Figure Task

**Figure 2.**
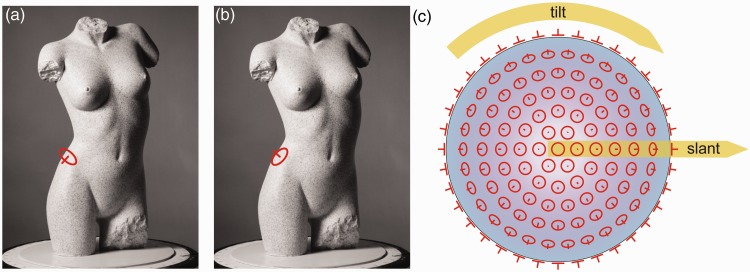
(a) An example of a setting of the gauge figure that is not visually consistent with the depicted object’s surface. (b) An example of a setting of the gauge figure that could possibly be considered as visually fitting the depicted object’s surface. (c) Examples of gauge figure settings on a sphere. The arrows indicate slant and tilt variations. The slant value is the angle between the line of sight and the normal on the tangent plane. The tilt value describes the direction of the slant. Each circle of gauge figures on the sphere thus possesses the same slant value, although a different tilt value.

The gauge figure (see also Koenderink, van Doorn, & Kappers, 1992) was similar to a thumbtack: It consisted of the outline of an ellipse with a line segment sticking out from the center of the ellipse. The long axis of the ellipse was kept constant on 40 pixels; the maximum length of the line segment measured 20 pixels. By manipulating a trackball, the shape and the orientation of the gauge figure could be adjusted. The task consisted of adjusting this gauge figure, superpositioned onto the depicted torso, so that it looked as if fitting in the scene. When having adjusted the gauge figure appropriately, it had to look like a circular figure painted onto the torso’s surface, with a line segment sticking out in the normal direction (Figure 2(b)). When the gauge figure was perceived to locally fit the surface, the participant clicked a button situated on the trackball. The attitude parameters (slant and tilt value; Figure 2(c)) as indicated by the gauge figure setting, were saved. Subsequently, the gauge figure appeared at another location on the picture.

The location of the gauge figure was selected randomly out of a pool of locations, one at a time. The pool of locations of the gauge figure was determined by the barycenters specified by a triangulation. The frontal pose (Pose 60) counted 528 locations; the dorsal pose (Pose 280) 483. Each picture was triangulated by superimposing a grid of equilateral triangles on top of the image plane within the traced contour of the depicted object. Participants were not aware of the triangulation when performing the task. Each participant ran three sessions for each of the 12 conditions (i.e., combinations of picture orientation × participant orientation, see Figure 1). Sessions per condition did not differ from each other, except for the order of the locations where the gauge figure appeared.

Viewing conditions were as follows. The picture was displayed in the center of a 21-inch flat computer screen (LACIE elektron 22 blue). The size of the picture measured 600 × by 800 screen pixels. The resolution of the screen was 1024 × 1280. The monitor was placed in a frontal-parallel position relative to the participant. The center of the picture was straight ahead with respect to the viewpoint of the participant. The eye-to-screen distance measured 50 cm. Head movements were restricted to a minimum by using a chinrest in the upright participant orientation (VF0) or by using a stabilizing pillow in the VF90 and VF270 participant orientation. Viewing was monocular, as previous research (Koenderink, van Doorn, & Kappers, 1994) has shown that compared with binocular viewing, monocular viewing increases the depth impression in pictures. The room was somewhat darkened, but the contours of the screen were still dimly visible.

### General Data Evaluation

**Figure 3.**
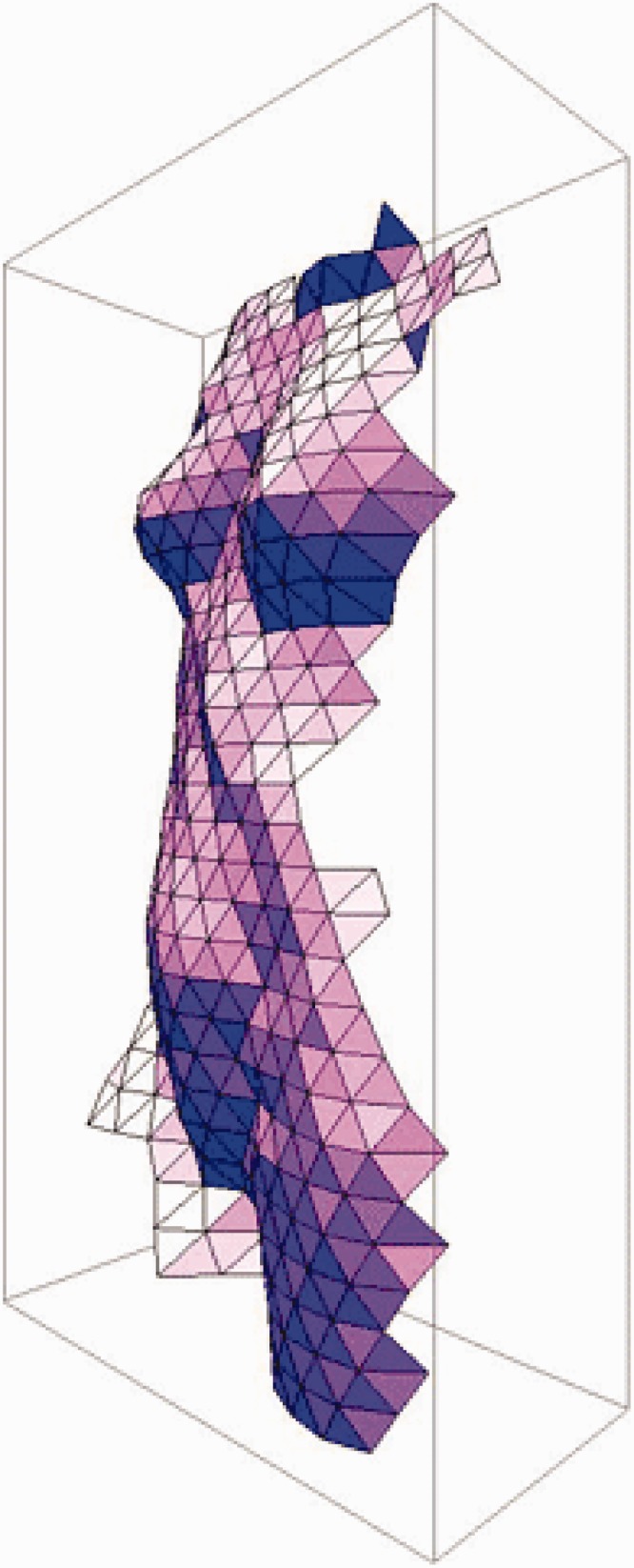
The reconstructed pictorial relief of the depicted object photographed in a frontal view (Pose 60) with the orientation of the picture, F0 and the orientation of Participant AD, VF90. This figure is also made available as animated movie in an online supplement.

The attitude settings, slant and tilt, were gathered for every condition. On the basis of the slant and tilt values collected over the entire area of the depicted object in one session, depth gradients were calculated at each probe’s position. Next, depth differences over the faces of the triangulation were computed. The mean of the depth values was set to zero, so as to eliminate a depth offset of the pictorial relief. Subsequently, depth values were obtained by using the least squares method to achieve the best fit with the collected attitude settings. Finally, the depth values based on attitude settings of each session were averaged over the three sessions per condition. For a more extensive explanation of the procedure by which a pictorial relief is constructed from the responses of the participant, we would like to refer to Koenderink et al. (1992). Figure 3 shows an example of a pictorial relief.

## Results and Discussion

### Analyses

**Figure 4.**
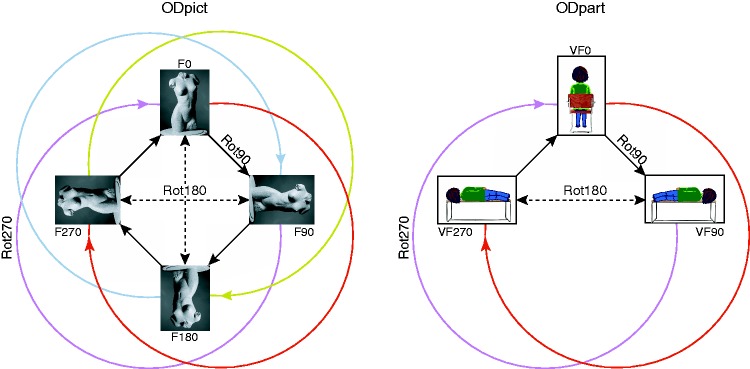
Graphical representation of the various comparisons within ODpict (difference between picture orientations) and ODpart (difference between participant orientations). The Rot-categories indicate the degrees by which the orientations differ with the black arrows, Rot90, the dashed arrows, Rot180, and the colored circular arrows, Rot270. Please note that the Rot0-categories are not included.

We compared the pictorial reliefs based on different picture orientations and participant orientations. An overview of all possible comparisons between the (reference and comparison) pictorial reliefs is presented in Table 2 in the Supplement.^2^ Each comparison can be described by a difference between picture orientations as well as a difference between participant orientations. Both the differences between picture orientations (ODpict) and the differences between participant orientations (ODpart) consist of the categories Rot0, Rot90, Rot180, and Rot270 (indicating the orientation difference in degrees; Figure 4).

 Quantitative comparisons of the depth values were conducted by means of (1) simple regression analyses and (2) multiple regression analyses. As explained later, both regression analyses involve components that allow to describe the (dis)similarity between each pair of pictorial reliefs by means of specific transformations.

1. The simple (*straight*) regression analyses relates the depth values of the comparison pictorial relief to the depth values of the reference pictorial relief (*z*_comp_ = *a* + *dz*_ref_, with *z* representing the depth values). As mentioned previously in Cornelis et al. (2003, 2009), the straight regression analysis accounts for possible depth scaling effects between the pictorial relief of the reference picture and the comparison picture. The slope of the regression line (*d* value) indicates the extent and the nature of the depth scaling found in the pictorial relief based on the reference picture with regard to the pictorial relief of the comparison picture. The pictorial relief of the reference picture can be similar, flattened, or deepened compared with the pictorial relief obtained from the comparison picture.
2. The multiple regression analysis (*z*_comp_ = *a* + *bx* + *cy* + *dz*_ref_, with *z* representing the depth values; with the image coordinates *x* and *y*, measured in screen pixels, referring to the positions on the image plane on which the depth values were calculated) takes into account not only the depth dimension but also the *x* and *y* dimension of the image plane. This way, the multiple regression analysis can reveal a depth scaling as well as a shear between the pictorial reliefs of the comparison picture and the reference picture. This multiple regression analysis can be considered as an affine transformation because of its geometrical properties: invariance of parallelism, collinearity, and ratios (for more information on the geometrical nature of the affine transformation, see Koenderink, van Doorn, Kappers, and Todd (2001); for information on the perceptual importance of the affine properties, see for instance Wagemans, Van Gool, Lamote, and Foster (2000). From now on, we will use the term “*affine regression*” when referring to the multiple regression as defined earlier.

Both the straight regression analysis and the affine regression analysis were carried out for each possible comparison of the pictorial reliefs, for each pose and participant separately.

In the next section, we will divide the comparisons in subsets in order to attempt to disentangle the respective importance of the environmental and the viewer-centered reference frame in shape perception. First, however, we will discuss the general effects of picture orientation and participant orientation on the shape percept of the depicted object.

### The Extent of (Dis)similarity Between the Pictorial Reliefs

#### General effects of picture orientation and participant orientation

To obtain general insight into the effects of picture and participant orientation on the shape percept of the depicted object, a statistical analysis was performed on the comparisons of the pictorial reliefs, with the coefficient of determination (*R*^2^ value^3^) obtained from the straight regression analysis as dependent variable, and Participant, Pose, the difference between picture orientations (ODpict) and the difference between participant orientations (ODpart) as independent variables.

 In this study, an *R*^2^ value obtained from the straight regression serves as a summarizing measure of the relationship between two pictorial reliefs. The height of the *R*^2^ value is indicative of the strength of the linear relationship between the depth values of the pictorial reliefs, and thus suggesting a higher or lower similarity between the pictorial reliefs within a comparison.^4^

Because there were several observations for each combination of ODpict and ODpart as well as a moderately balanced distribution of the observations over the various combinations and missing data points (e.g., the combination Rot0 picture orientation × Rot0 participant orientation did not exist in our study), we conducted a multilevel analysis with Participant as random variable and Pose, ODpict, and ODpart as fixed variables (for more background on multilevel regression, see Gelman & Hill, 2007).^5^ Significant main effects were observed for ODpict, F(3, 497) = 15.515, *p* < .0001, as well as for ODpart, *F*(3, 497) = 4.032, *p* = .008. For a graphical representation of the *R*^2^ values within ODpict and ODpart, see Figure 4. A Bonferroni post hoc test revealed that within ODpict, Rot180 was significantly different from Rot0 (*p* < .0001) and Rot90 (and of course Rot270; *p* < .0001): Clearly, Rot180 involved more dissimilarity between the pictorial reliefs compared with the other rotational categories. Interestingly, the Rot180 transformation was related to larger distortions when it concerned ODpict compared with ODpart. Within ODpart, Rot180 showed significant larger *R*^2^ values than Rot0 (*p* < .0001). In addition, Rot90 (and thus Rot270 as well) within ODpart demonstrated somewhat larger *R*^2^ values than Rot0 (*p* = .088). When the orientation of the participant stayed constant (Rot0 within ODpart), the effect of the difference between picture orientations (Rot90, and thus also Rot270, and Rot180) was larger compared with ODpart Rot90 (and Rot270) and Rot180. In contrast, when the picture orientation stayed constant (Rot0 within ODpict), and the participant orientation was varied (Rot90, Rot270, and Rot180 within ODpart), the similarity between the pictorial reliefs was considerable, suggesting that the variation within the participant orientation on itself did not lead to large effects on the shape percept.

Furthermore, the specific interplay between ODpict and ODpart was especially noticeable for Rot90 (and thus Rot270) and Rot180 within ODpict, and for Rot90 (and thus Rot270) within ODpart. When the differences between the participant orientations were identical to the differences between the picture orientations (or vice versa), the pictorial reliefs were more similar than when the differences between the picture and the participant orientations did not coincide (e.g., the Rot90 ODpart category within Rot90 ODpict, indicated by the yellow star in the second (blue) box from the right, Figure 4). We will discuss this further in the next section.

Besides the effects of ODpict and ODpart, there was also a main effect of Pose, *F*(1, 497) = 27.075, *p* < .0001. Pose 280 generally displayed higher *R*^2^ values than Pose 60 (mean *R*^2^ Pose 60; Pose 280 for A.D., .373; .562; for E.C., .319; .373).

**Figure 5.**
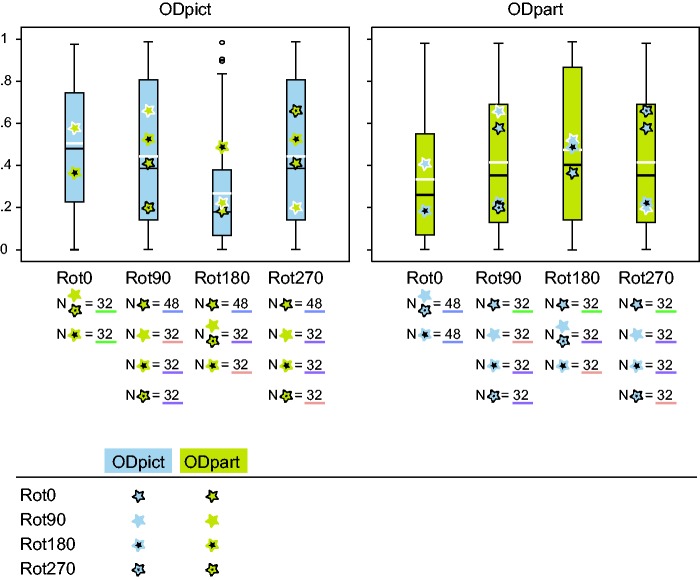
Box plots of the *R*^2^ values of the straight regression displayed for all rotational categories within ODpict (at the left) and ODpart (at the right). A black line indicates the median and a white line the mean. Outliers are symbolized by a circle. *N* refers to the number of comparisons; in total, there were 528 comparisons (132 comparisons × 2 poses × 2 participants). As one can deduce from the legend, the Rot0 category within ODpict refers to the comparisons with no change in picture orientation (Rot0) but with changes in participant orientation (ODpart; Rot90, Rot180, Rot270). Similarly, the Rot0 category within ODpart includes the comparisons with changes in picture orientation (ODpict; Rot 90, Rot180, Rot270) and no change in participant orientation (Rot0). The underlining in color refers to the various subsets: Subset 1, 2, 3 and 4. Please note that the sum of the means of ODpict and ODpart results in the same value—after all, both box plots concern the same data, which are visualized differently—and that all *R*^2^ values of the straight regression occur twice (i.e., ODpict Rot90 and Rot270; ODpart Rot90 and Rot270; ODpart Rot90 and Rot270, with only the Rot90 symbol visible since the symbols overlap, within ODpict Rot0 and Rot 180; ODpict Rot90 and Rot270 within ODpart Rot0 and Rot180). For reasons of clarity, we presented all *R*^2^ values in the box plots (for the motivation, refer to note 5, in which we explain the inclusion of all *R*^2^ values in the statistical analysis).

In conclusion, on the basis of these results, it is clear that both picture orientation and participant orientation play a role in shape perception. In the following, we will explore the contributions of the environmental and the viewer-centered reference frame in the results mentioned earlier (Figure 5).

#### General effects of the environmental and the viewer-centered reference frame

**Figure 6.**
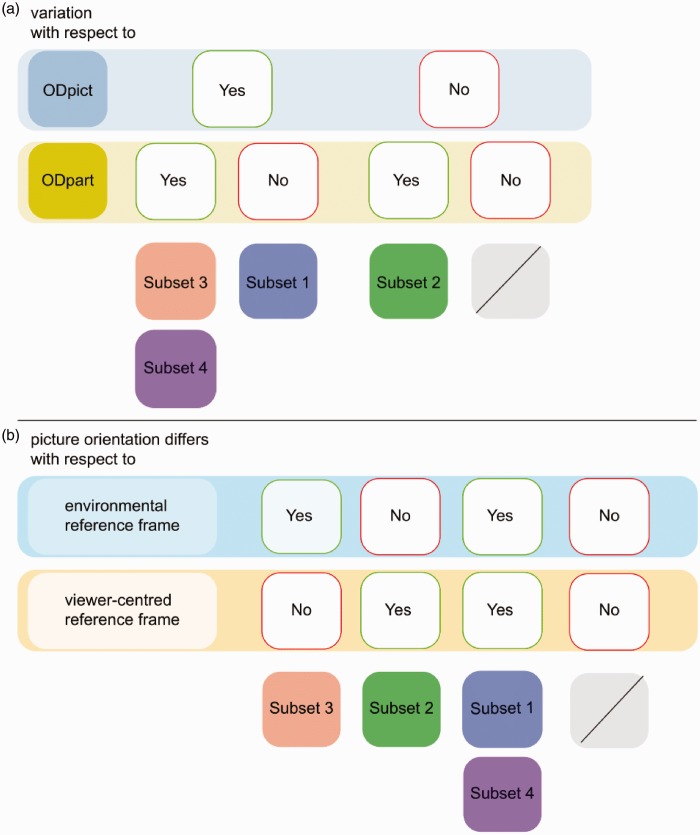
Overview of subsets (a) with regard to the differences between picture orientations (ODpict) and/or the differences between participant orientations (ODpart), (b) with regard to the environmental and the viewer-centered reference frame. As one can notice, each of the four subsets is indicated by a color; the gray boxes refer to the comparisons which we did not include in this study.

We divided the comparisons between the pictorial reliefs into four subsets. Every subset indicated a different relative importance of the environmental and the viewer-centered reference frame (Figure 6).

 The first subset consisted of comparisons for which the picture orientation was altered but not the participant orientation.^6^ Within these comparisons, the picture orientation thus differed with regard to both the environmental and the viewer-centered reference frame.

 In the second subset, the participant orientation was varied and the picture orientation stayed constant within the comparisons. In other words, the picture orientation changed only with respect to the viewer-centered reference frame.

 The third subset comprised the comparisons for which both the picture orientation and the participant orientation were varied to the same extent, meaning that the picture orientation differed with regard to the environmental reference frame only.

 Finally, the fourth subset included the comparisons not belonging to any of the three former mentioned subsets, with changes in both picture orientation and participant orientation and with the picture orientation differing with regard to the environmental as well as the viewer-centered reference frame.

**Figure 7.**
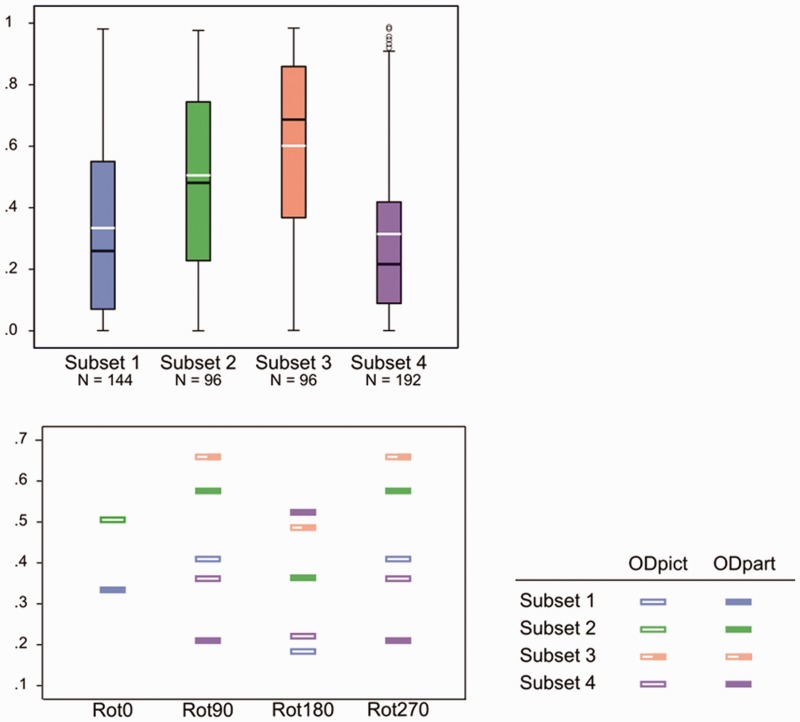
Above, box plots of the *R*^2^ values of the straight regression displayed by subset. The median is indicated by a black line, the mean by a white line. Outliers are symbolized by a circle. Below, the mean *R*^2^ values of the straight regression presented for all rotational categories (Rot0, Rot90, Rot180, and Rot270) of ODpict (difference between picture orientations) and ODpart (difference between participant orientations) within each subset. The Rot0 category can be considered as displaying the means of Subsets 1 and 2 (also indicated by the white lines in the box plots). ODpict and ODpart of Subset 3 are identical, as indicated by the half-filled rectangle. Please note that, since this analysis concerns the straight regression, the *R*^2^ values occur twice. For reasons of clarity, we chose to present all *R*^2^ values in the plots.

We conducted a multilevel analysis on the *R*^2^ values of the straight regression but now Participant, Pose, and Subset were independent variables (with Subset and Pose as fixed variables, and Participant as random variable). We found a main effect for Subset, *F*(3, 519) = 27.934, *p* < .0001. Subset 1 differed significantly from Subset 2 (*p* < .0001) and Subset 3 (*p* < .0001). Additionally, Subsets 2 and 3 were both significantly different from Subset 4 (*p* < .0001; *p* < .0001).^7^ On the basis of the box plots of the *R*^2^ values of the straight regression (Figure 7), one can clearly observe that Subsets 1 and 4 obtained the lowest *R*^2^ values indicating a weak (straight) relation between the pictorial reliefs (*R*^2^ Subset 1 = .334; *R*^2^ Subset 4 = .315). Subsets 2 and 3 demonstrated the highest *R*^2^ values (*R*^2^ Subset 2 = .505; *R*^2^ Subset 3 = .601). Apparently, picture orientations that were varied but kept a constant relationship with respect to the viewer-centered reference frame (Subset 3), resulted in minimal deviation between the pictorial reliefs. In addition, when only the participant orientation was varied and the picture orientation thus stayed the same with respect to the environmental reference frame (Subset 2), the pictorial reliefs were rather similar as well. Separating the influence of both reference frames, it thus seemed that neither the environmental (Subset 3) nor the viewer-centered reference frame (Subset 2) by itself affected the pictorial reliefs considerably (apart from possible depth scaling effects, see note 4). Furthermore, the pictorial reliefs within Subset 2 were more similar than the pictorial reliefs within Subset 1, suggesting that the effect of the difference in participant orientation was smaller than the effect of the difference in picture orientation. In addition, not only Subset 1 exhibited low *R*^2^ values; as mentioned earlier, Subset 4 demonstrated comparable *R*^2^ values. As shown in Figure 6(b), Subsets 1 and 4 both involved picture orientations that differed with regard to the environmental as well as the viewer-centered reference frame. These observations suggest that the interplay between both reference frames is important in explaining the distorting effects of orientation on shape perception.

 More specifically, when the picture orientation differs with respect to only one reference frame, the effect on the shape percept is smaller than when the picture orientation differs with respect to both the viewer-centered and the environmental centered reference frame.

#### Specific interplay between picture orientation and the environmental and viewer-centered reference frames

**Figure 8.**
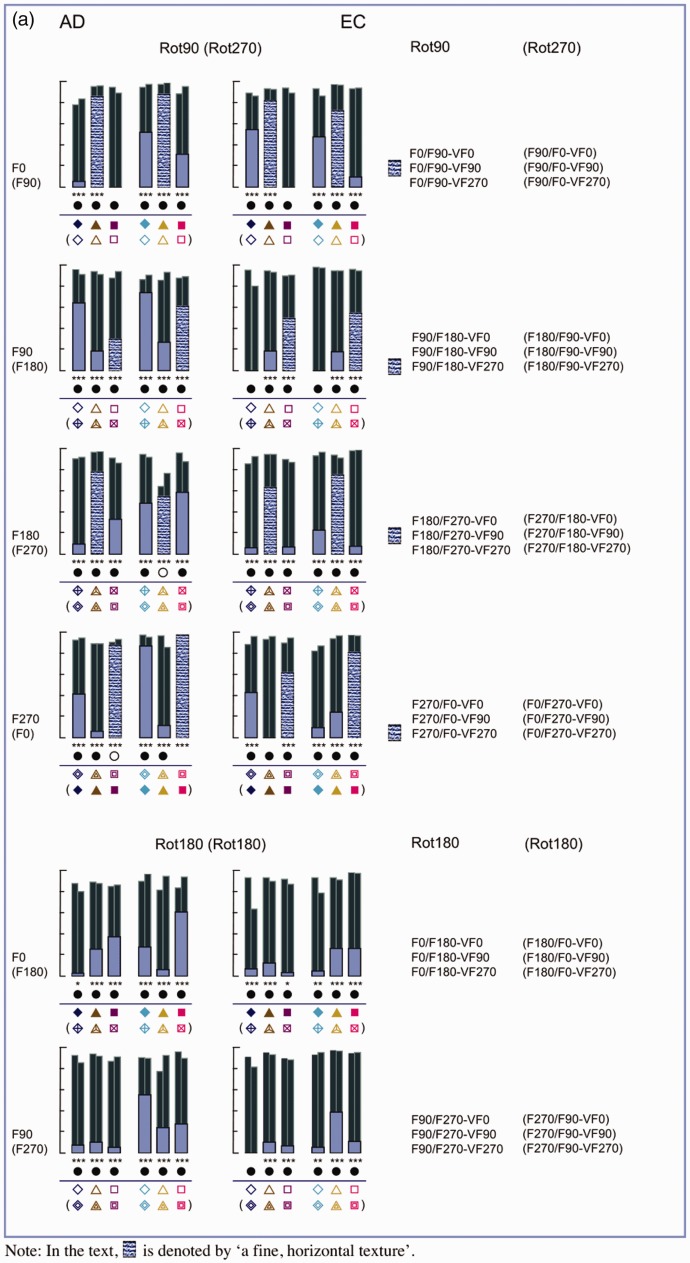

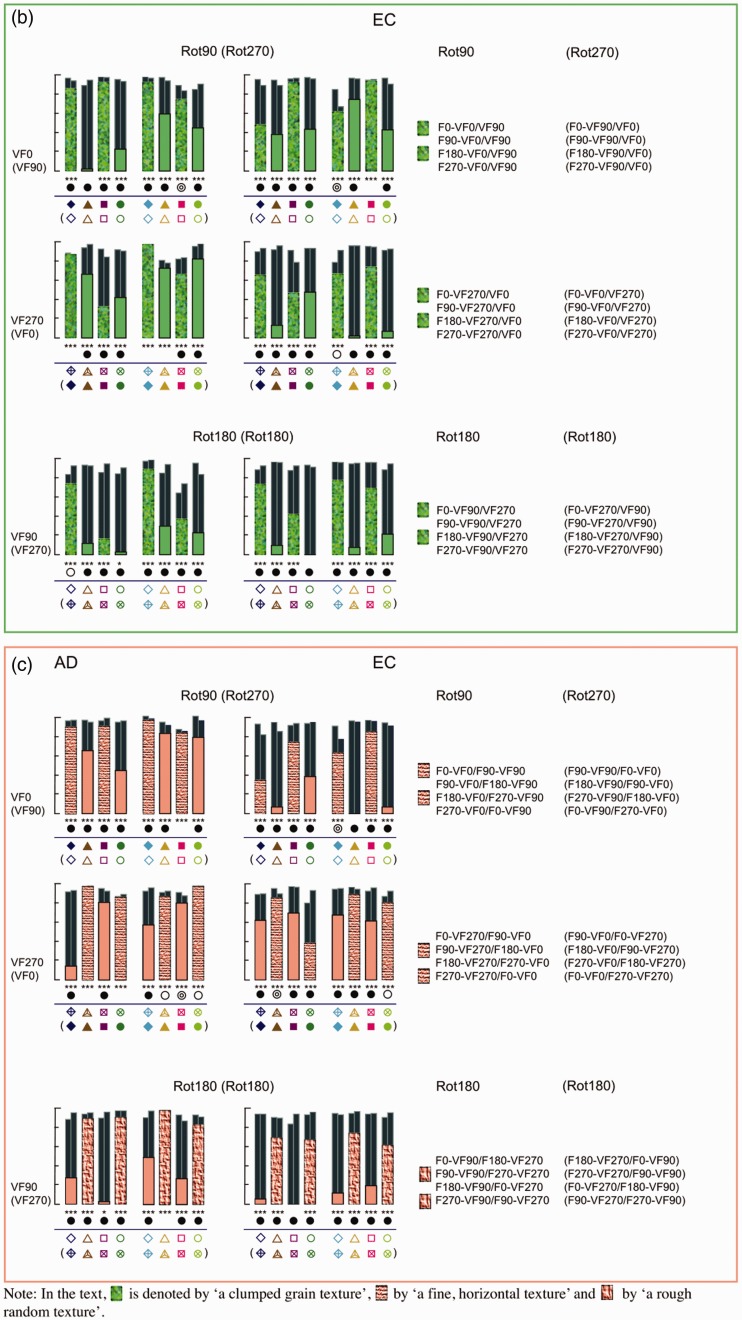

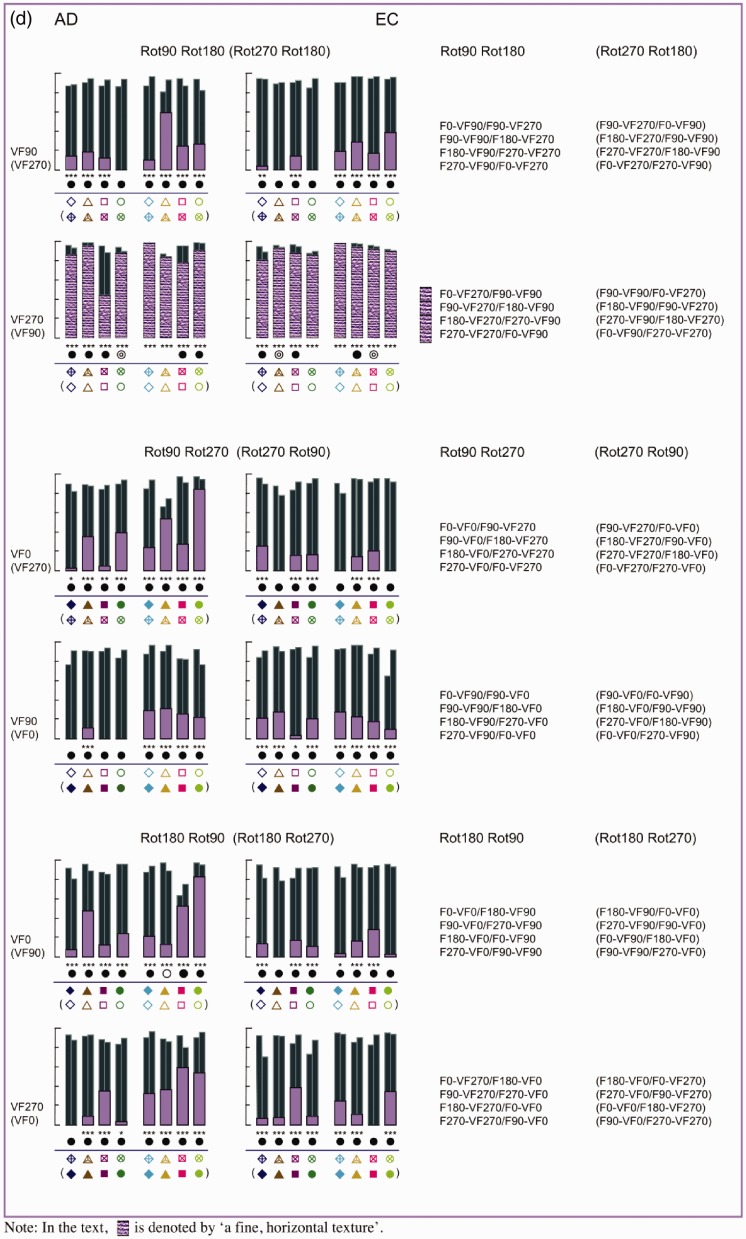
(a) Bar charts of the *R*^2^ values of the straight (blue bars) and affine (dark bars) regression of Subset 1, with the rotation categories above the bar charts referring to ODpict. (b) and (c). Bar charts of the *R*^2^ values of the straight (green or pink bars) and affine (dark bars) regression of Subset 2 (b), with the rotation categories above the bar charts referring to ODpart, and Subset 3 (c), with the rotation categories referring to both ODpict and ODpart. (d). Bar charts of the *R*^2^ values of the straight (purple bars) and affine (dark bars) regression of Subset 4, with the rotation categories above the bar charts referring to ODpict and ODpart, respectively. Figure 8(a) to (d) shows bar charts displaying the *R*^2^ values of the straight (light colored bars) and affine (dark colored bars) regression for the comparisons of Subsets 1–4, for Participant A.D. (at the left) and Participant E.C. (at the right).

Figure 8 shows the individual *R*^2^ values of the straight and the affine regression obtained from the comparisons between the pictorial reliefs within each subset. First, we will continue to focus on the straight regression (with the *R*^2^ values represented by the thick bars and a different color for each subset). Later in this article, we will elaborate on the affine regression (with the *R*^2^ values represented by the small, dark bars).

**Figure 9.**
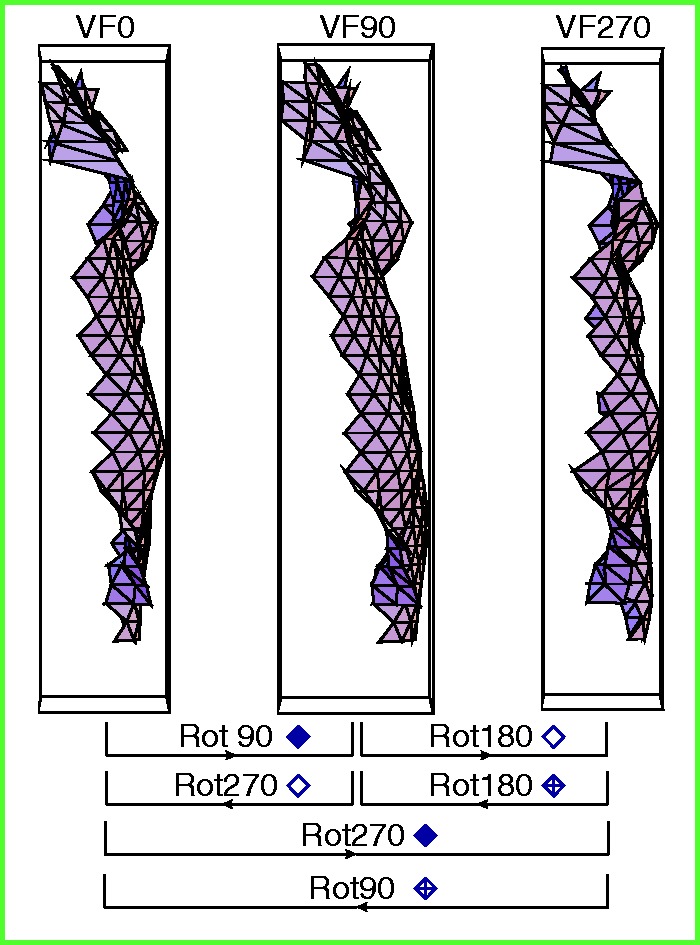
Side views of the pictorial reliefs, based on the F0 picture orientation of the photograph of the frontal pose of the torso, computed from the gauge figure settings of AD. The comparisons belong to Subset 2 and can be described by a constant relationship of the picture orientation with the environmental reference frame (see comparisons indicated by Δ in the text). It is easy to note that the pictorial reliefs are similar to very similar.

 As we mentioned earlier, for every comparison, there was an inverse comparison that resulted in the same *R*^2^ value for the straight regression (for the inverse comparisons, see the notations between brackets). In the following, we will only mention one of the two comparisons related to that same *R*^2^ value, that is, the first comparison with the lowest difference between the picture orientations followed by the lowest difference between the participant orientations. For instance, we will mention the comparisons of the Rot90-category but not the inverse comparisons belonging to the Rot270-category.

 To point at a specific comparison, we will use an abbreviated notation: “/” refers to the comparison between reference and comparison; “-” refers to the combination of picture orientation and participant orientation in either the reference or the comparison or both. For instance,“F0/F90-VF90” then indicates the comparison (of Subset 1) between the pictorial reliefs based on pictures differing by 90°, with reference picture F0 and comparison picture F90, and participant orientation VF90. Another instance, “F180-VF0/F0-VF90” is the comparison (of Subset 4) with reference picture orientation F180 and reference participant orientation VF0; the comparison picture orientation is F0 and the comparison participant orientation is VF90—the orientations between pictures thus differ 180°; the orientations between participants differ 90°. Furthermore, in order to facilitate reading, the comparisons we refer below are indicated by a specific texture in the bar charts.

As one can notice from the bar charts of Subsets 1–3, both participants generally showed lower *R*^2^ values for Rot180 compared with Rot90 (mean *R*^2^ Rot180; Rot90 for A.D., Subset 1, .242; .482; Subset 2, .354; .639; Subset 3, .585; .776; for EC, Subset 1, .125; .337; Subset 2, .373; .513; Subset 3, .388; .542). In other words, the Rot180 transformation, with regard to picture orientation (Subset 1), participant orientation (Subset 2), or both (Subset 3), showed larger effects compared with the Rot90 (and thus Rot270) comparisons (see also Figure 7, displaying the mean *R*^2^ values of every rotational category within ODpict and ODpart for each subset).

However, the *R*^2^ values of the straight regression also differed considerably *within* the rotational categories. Since the difference between picture and participant orientations could evidently not account for the differences in *R*^2^ values within the rotational categories—after all, the differences between picture and participant orientations were the same within each rotational category—we explored the specific comparisons in more detail in order to gain more insight into other contributing factors. A.D. and E.C. differed somewhat concerning the extent of similarity between the pictorial reliefs, with A.D. generally exhibiting higher *R*^2^ values than E.C. (mean *R*^2^ for A.D., .467; for E.C., .346). In addition, from time to time, A.D. and E.C. showed slightly deviating trends suggesting that in some cases, the perceptual influence of the environmental and the viewer-centered reference frame might have differed a little. Having said that we will particularly focus on the obvious, common trends.

#### The correspondence of the picture orientations with the environmental and the viewer-centered reference frame

Coincidence of the picture orientation with the environmental and the viewer-centered reference frame seemed to be of great importance to explain some of the results. The *R*^2^ values presented by the bars with a fine, horizontal texture (see Figure 8(a), (c) and (d)) all result from comparisons that involved a particular correspondence of the picture orientations with the environmental and the viewer-centered reference frame. One can easily note that the *R*^2^ values corresponding with these comparisons were in almost all cases higher than the *R*^2^ values of the other comparisons in the same bar chart. In Subset 3, the specific comparisons (F0-VF0/F90-VF90; F180-VF0/F270-VF90; F90-VF270/F180-VF0; F270-VF270/F0-VF0^8^) included the F0 or the F180 picture combined with the VF0 participant orientation; the other orientation of the picture (F90 or F270) was upright or upside down in relation to the participant orientation (VF90 or VF270). The upright or upside down relationship between one of the picture orientations (F0 or F180) and the environment as well as the participant orientation (i.e., VF0) was thus identical to the upright or upside down relationship between the other picture orientation and the participant orientation.

**Figure 10.**
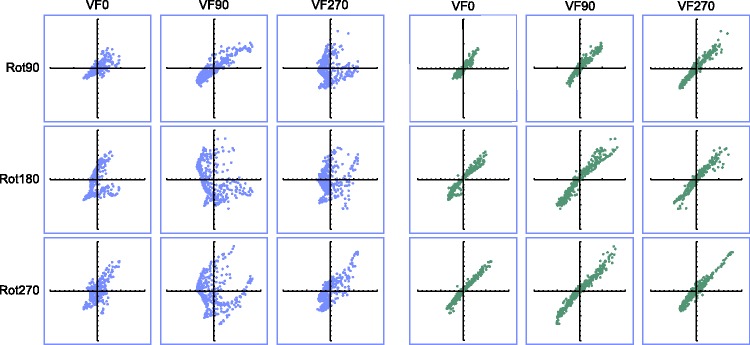
At the left, scatter plots of the raw depth values obtained from the original picture F0 (*x* axis) vs. the raw depth values obtained from the comparison picture F90, F180, or F270 (*y* axis). At the right, scatter plots of the affinely corrected depth values from the original picture F0 (*x* axis) vs. the raw depth values obtained from the comparison picture F90, F180, or F270 (*y* axis). The axes range from −200 to 200 pixels. Rot90, Rot180, and Rot270 indicate the comparisons between the pictorial reliefs based on pictures differing by 90°, 180°, and 270°, respectively (Subset 1). The second (raw) scatter plot of the first row and the third (raw) scatter plot of the third row display the depth values of the comparisons (^) which could be specified by correspondences of the picture orientations with the environmental and the viewer-centered reference frame. VF0, VF90, and VF270 refer to the participant orientations. The depth values shown were gathered from the photograph of the frontal view of the torso (Pose 60), from Participant EC.

Some specific comparisons of Subset 1 (F0/F90-VF90^; F90/F180-VF270; F180/F270-VF90; F270/F0-VF270^; for the comparisons with ^, see also Figure 10) and Subset 4 (F0-VF270/F90-VF90; F90-VF270/F180-VF90; F180-VF270/F270-VF90; F270-VF270/F0-VF90^9^) demonstrated that the correspondence of the F0 or the F180 picture with the VF0 participant orientation was, however, of minimal importance: The inherent correspondence between the orientation of the picture (either F0 or F180) and the environmental reference frame seemed to overrule the influence of the participant orientation.

#### The correspondence of the picture orientation with the environmental reference frame

In the previous section, we already touched upon the importance of the environmental reference frame over the viewer-centered reference frame when the comparisons encompassed the F0 or F180 picture orientation. In some comparisons of Subset 2, the inherent strong correspondence of picture orientations F0 and F180 with the environmental reference frame even seemed to be sufficient to result in high similarities between the pictorial reliefs.

The distorting influence of the variation in participant orientation seemed to be generally suppressed in the comparisons indicated by a clumped grain texture in Figure 8(b) (i.e., F0-VF0/VF90 Δ, F180-VF0/VF90, F0-VF270/VF0 Δ, F180-VF270/VF0, F0-VF90/VF270 Δ, F180-VF90/VF270, F0-VF90/VF270 Δ, F180-VF90/VF270), all including the F0 or F180 picture orientation that had been looked at from two different participant orientations (for the comparisons with Δ, see also the pictorial reliefs in Figure 9).

Especially the high similarities between the pictorial reliefs occurring in Rot180 are surprising, as this rotational category is generally associated with rather large effects on the shape percept. A possible interpretation concerning the high *R*^2^ values of F0-VF90/VF270 or F180-VF90/VF270 could be that the F0 or the F180 picture orientation was in-between the VF90 and the VF270 orientation of the participant. However, considering the high *R*^2^ values related to the other, above-mentioned comparisons also including F0 or F180, it seemed more logical to assume that the strong congruence of the F0 or F180 picture orientation with the environmental reference frame explained the high similarities between the pictorial reliefs. Furthermore, clearly, the importance of the canonical orientation of F0 in obtaining high *R*^2^ values was contradicted by the comparisons containing F180, which also revealed high *R*^2^ values.

#### The correspondence of the picture orientations with the viewer-centered reference frame

Earlier, we have already noted that the general distortion in shape perception was rather small when the picture orientation varied to the same extent as the participant orientation (Subset 3; see Figure 7). The two components of each comparison belonging to Subset 3 possessed the same correspondence with the viewer-centered reference frame. Some comparisons revealing higher *R*^2^ values than others could be characterized by a correspondence of the picture orientations with the environmental and the viewer-centered reference frame (see earlier; denoted by a fine, horizontal texture in the bars). In addition, both parts of the comparisons F90-VF90/F270-VF270 and F270-VF90/F90-VF270 (see the bars with a rough random texture in Figure 8 (c)), also exhibiting high *R*^2^ values, comprised an upright or upside down picture orientation with regard to the participant orientation.

 Summarizing, high similarities between pictorial reliefs were obtained for comparisons with:

‐ a correspondence between the picture orientations and the viewer-centered reference frame on the one hand and the environmental reference frame on the other hand, on the condition that one of the picture orientations was upright (F0) or upside down (F180) according to the environmental reference frame (fine, horizontal texture; Subsets 1, 2, 3 and 4).
‐ a correspondence between the picture orientation and the environmental reference frame, on the condition that the picture orientation involved F0 or F180, that is, the upright or upside down orientation with regard to the environmental reference frame (clumped grain texture; Subset 2).
‐ a correspondence between the picture orientations and the viewer-centered reference frame, on the condition that the picture orientation was upright or upside down with regard to the participant orientation (rough random texture; Subset 3).

All correspondences mentioned earlier included an upright or upside down picture orientation with regard to the environmental or the viewer-centered reference frame.

Obviously, the upright or upside down oriented picture involved an upright or upside down oriented torso. In the discussion section, we will discuss the importance of the upright or upside down orientation of the torso in more detail.

Having described the general effects of the correspondences of the picture orientation with one or both of the frames of reference, we will now briefly focus on the importance of the lighting direction. When varying the orientation of a picture by rotating it, the position of the light source in the picture obviously varies with it^10^—not with regard to the picture’s (intrinsic) reference frame, but with regard to the extrinsic frames of reference. Although some variation in the data might have been related to other variables, such as the different lighting directions when rotating or mirror-reflecting a picture, the differences between the pictorial reliefs could be largely ascribed to the transformation itself, as reported before (Cornelis et al., 2009). The present study enabled us to examine the effect of the position of the light source more closely. To this end, we compared the *R*^2^ values of comparisons that are largely identical, but by interchanging F90 by F270 and VF90 by VF270 included different relationships between the lighting direction and the environmental or the viewer-centered reference frame^11^ (see Table 3 in the Supplement). Examining the *R*^2^ values within these particular pairs of comparisons, it seems that the participants applied different strategies to perceptually handle the relationship between the lighting direction and the environmental and/or the viewer-centered frame of reference. More specifically, considering the lighting from above,^12^ Participant A.D. seemed to generally treat the left as more similar to the top and the right as more similar to the bottom (see pairs of comparisons 1, 4, 10, 11, 12, and 16 in Tables 3 and 4 in the Supplement). Participant E.C. demonstrated less distinct and less consistent trends. With regard to the top lighting in the original photograph F0, the right was found to be more related to either the top or the bottom, dependent on the specific pairs of comparisons (see pairs of comparisons 1, 9, 10; 4, 12; 11, 16 in Tables 3 and 4 in the Supplement).

The interindividual differences as well as the inconsistency within the participants’ data (especially of E.C.) may seem surprising considering the general importance often attributed to the light-from-above prior (e.g., Benson & Yonas, 1973; Kleffner & Ramachandran, 1992; Mamassian & Goutcher, 2001; Ramachandran, 1988; but see Adams, Graf & Ernst, 2004; Morgenstern, Murray & Harris, 2011; Proulx, 2014, for putting the robustness or the hard wiredness of the light-from-above prior into perspective). One has to bear in mind, however, that altering the orientation of the picture and thus, consequently, the position of the light source with respect to the environmental or the viewer-centered reference frame does not involve variations in the shading pattern on the depicted object caused by different lighting conditions (see e.g., Koenderink, van Doorn, Christou, & Lappin, 1996; Todd, Koenderink, van Doorn, & Kappers, 1996, on the effect of different lighting conditions on pictorial perception). Although we would like to assert that the *R*^2^ differences of Participant A.D., generally demonstrating the top orientation of the top lighting as perceptually more similar to the left orientation than to the right, may be to some extent related to the light-from-left above prior, it is obvious that the present study did not have enough participants to make strong claims concerning (interindividual) perceptual strategies with regard to the relationship between the lighting direction and the environmental or the viewer-centered reference frame. Future research is warranted to further investigate the effect of the lighting direction related to different orientations of the picture with respect to the environment or the participant.

#### The nature of the dissimilarities between the pictorial reliefs

Previously, we examined the extent of (dis)similarity between the pictorial reliefs of the comparisons. In this section, we investigate the nature of the dissimilarities between the pictorial reliefs.

As we described earlier, we considered the fit of the straight regression as a measure of the (dis)similarity between the pictorial reliefs. Geometrically, the straight regression accounts for a depth scaling between the pictorial reliefs. Since the *R*^2^ values of the straight regression were from time to time very weak to moderate, a depth scaling (determined by the weight of the depth values of the reference picture, “d”) did not seem to be sufficient to explain for the differences between the pictorial reliefs.

When taking into account not only the depth values of the reference picture but also the image coordinates, the *R*^2^ values of the (affine) regressions were highly significant for all subsets (*p* < .0001). Furthermore, a significant gain in proportion of explained variance was usually obtained when comparing the affine regression analysis to the straight regression analysis (*p* < .01; symbolized by a black, filled, or open circle in Figure 8). The better fit of the affine regression compared with the straight regression is also clear from Figure 10, presenting typical scatter plots representing the raw depth values as well as the affinely transformed depth values of the reference picture versus the observed depth values of the comparison picture: Compared with the raw scatter plots, the affinely corrected scatter plots demonstrate strong linear trends. This suggests that in order to transform the pictorial relief of the reference picture toward the pictorial relief of the comparison picture, an additional shear (determined by the weights of the image coordinates, “b” and “c”, in the affine regression) was necessary. Furthermore, it is clear that the clusters (previously shown to correspond with specific surface areas on the depicted object; see Cornelis et al., 2003) displayed in the raw scatter plots nearly completely vanished after the affine transformation, suggesting that one global shear was enough to explain for (most of) the differences.

Next, we discuss the shears obtained from the affine regressions for every comparison between the pictorial reliefs.

 The shears were calculated from the weights *b* and *c* of the image coordinates *x* and *y* in the affine regression model; the arctangent of the ratio of the weights *c* and *b* determines the direction of the shear; a measure for the magnitude of the shear was limited between 0 and 1 by calculating sin{arctan[√(*b^2^*+ *c^2^*)]} (for more information on the computation of the shears, see Koenderink et al., 2001).

 Before we move on to discuss some observations concerning the direction and the magnitude of the shears, first some explanation on how to read the polar plots. The shear symbol indicates the direction and the magnitude of the shear and can be considered as the end point of a rod that starts at the middle of the graph. Shear symbols situated at the left of the polar plot indicate that the right part of the pictorial relief of the reference picture needed an attitude change toward the front in order to fit the pictorial relief of the comparison picture; shear symbols positioned in the lower half of the plot imply that the upper part of the pictorial relief of the reference picture needed an attitude change toward the front in order to become the pictorial relief of the comparison picture.

In addition to the differences within *R*^2^ values, suggesting that A.D. and E.C. might perhaps use slightly different strategies from time to time, we also observed interindividual differences concerning the magnitude and the direction of the shears, especially in Subset 3. Moreover, the overall magnitude of the shears was somewhat higher for E.C. than A.D. (Table 1). Nevertheless, despite these interindividual differences, the qualitative trends were generally much the same for the two participants. Overall, there was clearly an effect of the rotational categories (Rot90, Rot180, and Rot270) on the direction of the shear in each subset: Depending on the degree of rotation between the picture orientations and the participant orientations, the shears behaved differently, as discussed next.

### Viewpoints-From-Above Defined by the Environmental and the Viewer-Centered Reference Frame

**Figure 11.**
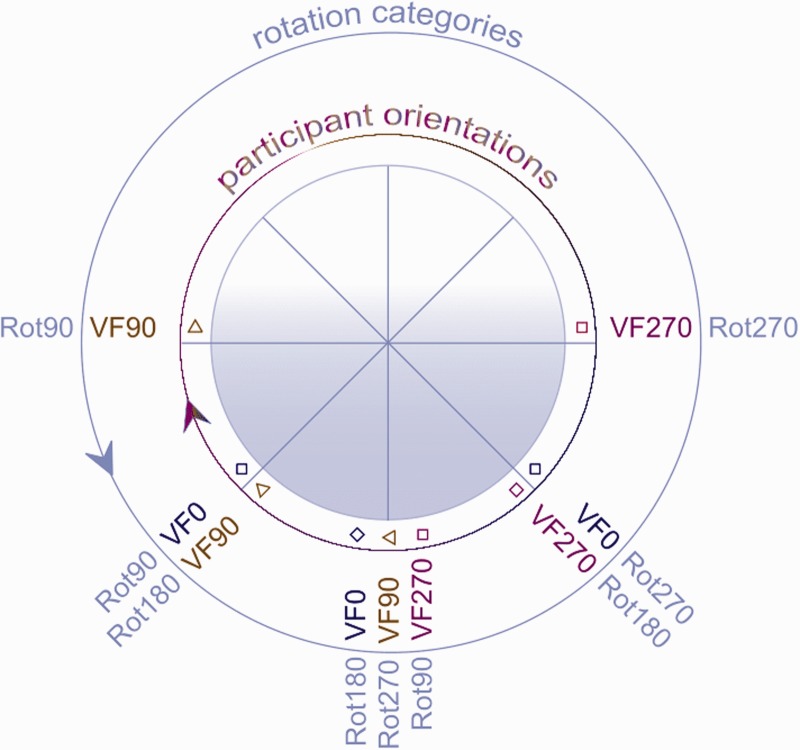
A schematic summary of the shears of Subset 1. The shears are specified by the rotation category (with regard to ODpict) as well as by the participant orientation. The symbol depicted in the inner circle refers to the participant orientation, generalized over all reference picture orientations. Please note that this schematic drawing shows the shears of Subset 1 by approximation. Figure 12(a) shows the actual shears.

**Figure 12.**
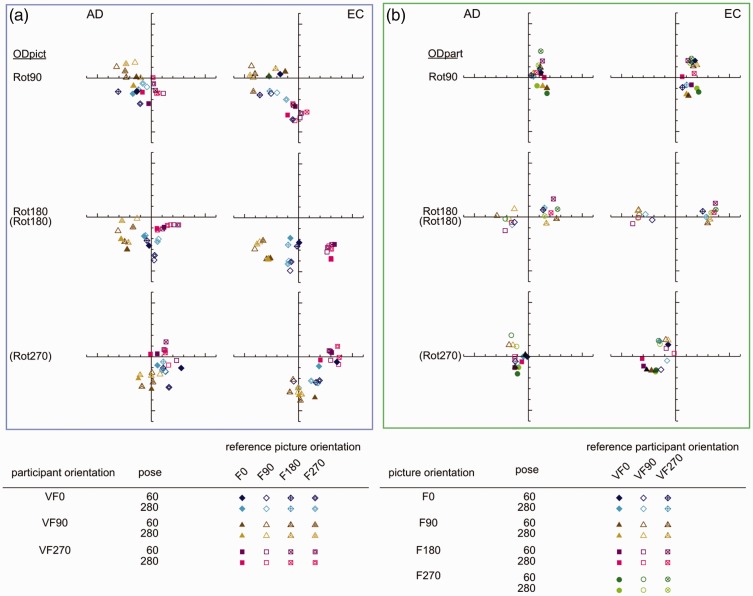

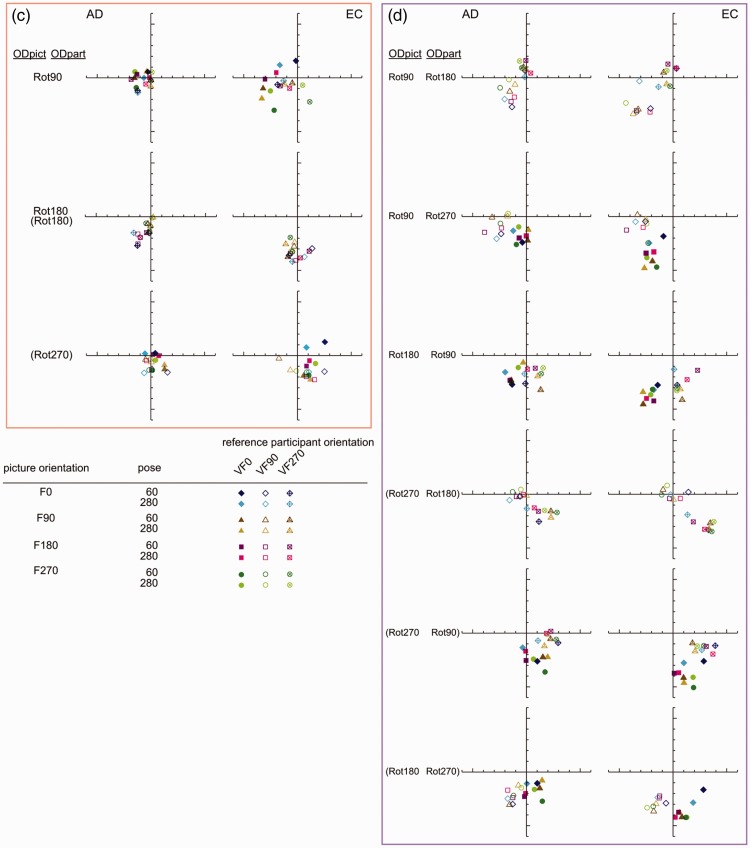
(a) to (d) Polar representations of the shears between the pictorial reliefs are obtained from AD (at the left) and EC (at the right). The *x* and *y* axis refer to the horizontal and the vertical dimensions of the image plane, whatever the reference orientation of the picture or participant may be. Both axes range from .6 to −.6, thereby only presenting a section of the total range (−1 to +1).

Let us first have a closer look at the shears of Subset 1^13^ (see Figure 11 for a schematic summary and Figure 12 (a) for details). Both the shear symbols of Rot90 and Rot180, and the shear symbols of Rot180 and Rot270, differ by approximately 45°. Within each rotation category, one can also easily notice a turn of about 45° between the VF90 and the VF0 clusters of shear symbols and between the VF0 and VF270 clusters of shear symbols.

Remarkably, the shear symbols corresponding with the various participant orientations did not differ by 90° as could be expected if the participant orientation would have a maximum effect; after all, the shear would be rotated to the same extent as the participant.

We think the key to understanding the behavior of the shears and the 45° differences between the clusters is to regard the shears as combinations of slants generated by a viewing-from-above bias (e.g., Mamassian & Landy, 1998; Troje & McAdam, 2010). Let us explain this.

This study examined the comparisons between pictorial reliefs, meaning that there is no common, general reference pictorial relief. Therefore, a shear between two pictorial reliefs can best be considered as the combination of the slant of the pictorial relief of a reference picture with the slant of the pictorial relief of a comparison picture. Let us suppose that, in accordance with the viewpoint-from-above prior, which assumes that humans have a tendency to perceive objects as if they were looked at from above, the bottom part of the depicted object is slanted to the front in every instance of looking at a picture.^14^ Exploring the viewpoint-from-above prior in combination with our data, we noticed that it was not enough to only include slants induced by the viewpoint-from-above which corresponded with the participant orientation; the environmental reference frame had to participate evenly in order to result in the observed shears.

**Figure 13.**
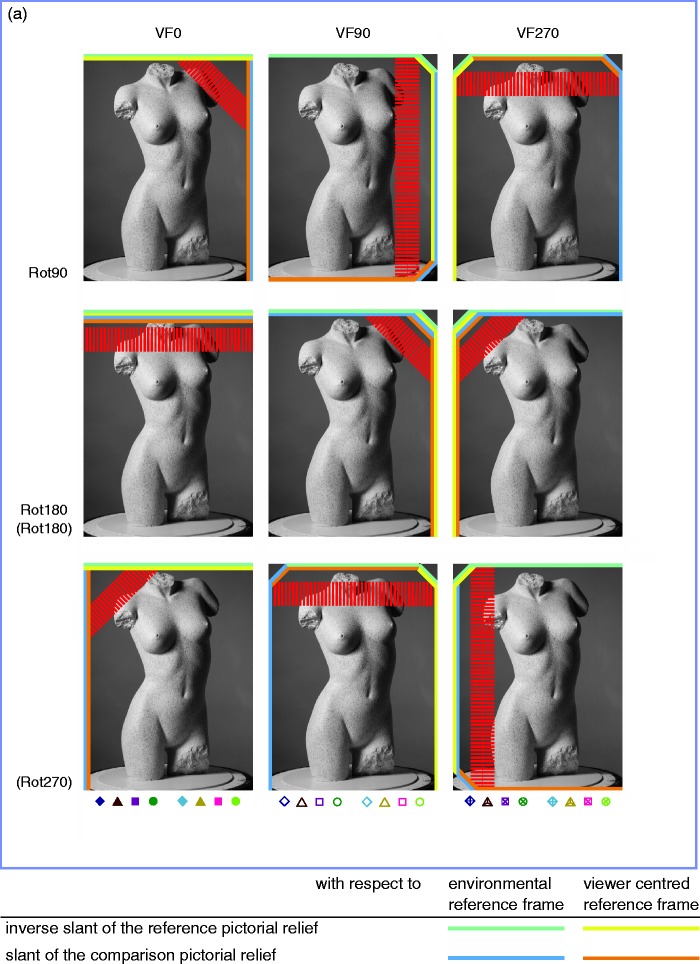

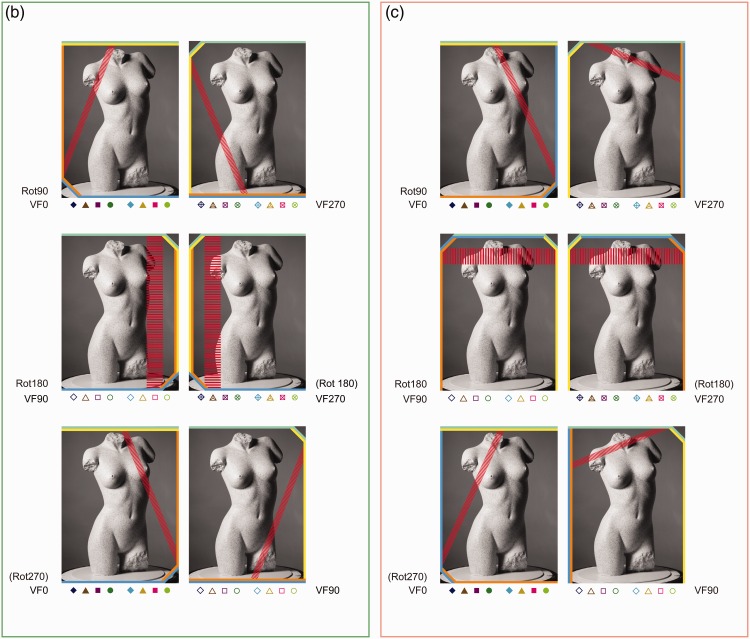

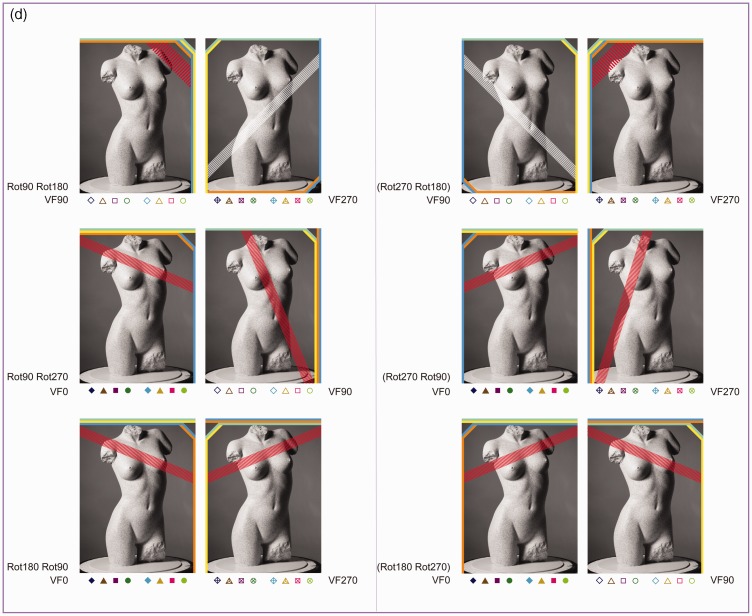
(a) Representation of the slants generated from the viewpoint-from-above, and the corresponding hypothesized shears; Subset 1. (b) and (c) Representation of the slants generated from the viewpoint-from-above, and the corresponding hypothesized shears; Subset 2 (b); Subset 3(c). (d) Representation of the slants generated from the viewpoint-from-above, and the corresponding hypothesized shears; Subset 4. Figure 13(a) to (d) shows the representation of the (frontward) slants, which we assumed to result from a viewpoint-from-above. The viewpoint-from-above was defined with respect to the environmental reference frame as well as the viewer-centered reference frame (see lines in color in legend). The inverse slant related to the reference picture—inverse because we considered the transformation that occurred from the pictorial relief of the reference picture toward the pictorial relief of the comparison picture—and the slant related to the comparison picture, were concatenated to result in the hypothesized shear, represented by the red striped line. The direction of the stripes indicates the direction of the (hypothesized) shear; the width suggests its magnitude. The stripes in white demonstrate the combinations of slants that led to a hypothesized shear of zero. Please note that the F0 picture orientations displayed in this figure were chosen arbitrarily; within each category of comparisons the combinations of slants were the same for all comparisons (for an overview of the comparisons within each rotation category, see the right of Figure 8).

 To clarify the viewpoint-from-above interpretation further, let us take the comparison F0/F90-VF90, belonging to Rot90, Subset 1, as an example. This comparison is represented by the second picture (column of VF90) in the first row (row of Rot90) of Figure 13 (a).

 Following the viewing-from-above assumption, we propose that the bottom of the picture is slanted to the front. Because we assume the shear transformation between the pictorial reliefs to be the result of the viewing-from-above bias when looking at a picture, we here use “picture” instead of “pictorial relief.”

 As mentioned earlier, we suggest that the viewing-from-above bias is determined not only with respect to the viewer-centered reference frame but also with respect to the environmental reference frame. This implies that, for the F0/F90-VF90 comparison, the left side of the reference picture F0 is regarded as the bottom with respect to the viewer-centered reference frame (VF90); the environmental reference frame assigned the frontward slant to the bottom of the reference picture F0. Because we considered the shear transformation of the reference pictorial relief toward the comparison pictorial relief, we inverted the slants of the reference picture (resulting in the yellow line at the right side of F0 and the cyan line at the top). The total of these two (inverse) slants resulted in the (inverse) viewpoint-from-above slant of the reference picture (yellow—cyan line at the right top corner).

 The viewpoint-from-above slant of the comparison picture (F90) was defined as follows. The concatenation of the frontward slant of the bottom of the F90 picture orientation (equivalent to the right side of F0; blue line) with regard to the environmental reference frame, with the frontward slant of the bottom of the F90 picture orientation with respect to the VF90 participant orientation (equivalent to the bottom of F0; orange line) resulted in a frontward slant of the bottom right corner (orange-blue line).

 Subsequently, the hypothesized shear (represented by the line with the red stripes) was determined as the concatenation of the (inverse) slant of the reference picture and the slant of the comparison picture. The hypothesized shear implied an attitude change of the right side of the picture to the front, and matched the observed shear (Figure 12 (a)) perfectly.

Also the other comparisons within Subset 1 showed concatenations of viewpoint-from-above slants that fitted the shear data extremely well (Figures 13(a) and 12(a)). Therefore, we believe the viewpoint-from-above interpretation to be valid and meaningful, and even more so since this viewpoint-from-above interpretation suited the shear data of the other subsets quite well (see below).

Before moving on to discuss the other subsets, we would like to note that from this analysis, it is not entirely clear if there is a mechanism involved that compensated for the participant orientations deviating from the upright (VF0) orientation, or that the viewpoint-from-above is simply determined by the two reference systems, the environmental and the viewer-centered reference frame. However, we are inclined to believe that the latter is the case. The viewpoint-from-above interpretation appears to be the most parsimonious and fitted the shear data of the other subsets as well. Moreover, the shears related to the VF0 participant orientations, for which the viewer-centered reference system clearly coincided with the environmental reference frame, showed similar properties (i.e., magnitudes and directions) compared with the shears related to VF90 and VF270. If a compensation mechanism were involved, then we would expect some deviations in magnitudes or directions between the VF0 clusters of shears on the one hand and the VF90 and VF270 clusters on the other hand. In addition, the environmental up can be defined in different ways: by the sense of gravity or by the perception of the outer world including the up-down direction. In this study, we did not disentangle these two possibilities, and therefore, we cannot answer which of the two affected the shears (most strongly).

As mentioned earlier, a similar line of thought can be followed for Subset 4 which showed shears that fitted the viewpoint-from-above interpretation well (Figures 12(d) and 13(d)). Apart from Rot180, the shears of Subsets 2 and 3 (Figure 12(b) and (c)) were not only considerably smaller than the shears of Subsets 1 and 4 but also showed less consistency and, at first sight, formed thus less strong evidence for the viewpoint-from-above interpretation. However, the slants that were generated by the viewpoints-from-above in Subsets 2 and 3 (especially for Participant A.D.), contradicted one another to a lesser or larger extent (Figure 13(b) and (c)), resulting in smaller shears. Contradicting slants in Figure 13 indicate similar viewpoint-from-above slants in the pictorial reliefs,^15^ and high similarity between pictorial reliefs. The smaller shears in Subsets 2 and 3, and the corresponding viewpoint-from-above interpretation, are consistent with our explanation in terms of the constancy of the picture orientation with respect to either the environment (Subset 2) or the participant (Subset 3; see Figure 6 and General effects of the environmental and the viewer-centered reference frame section). Obviously, the specific variations in participant orientation and picture orientation within each subset are inextricably linked to the specific viewpoint-from-above slants.

In addition to the general effects of the viewpoint-from-above slants, we briefly mention some remaining observations in the following. For example, compared with the comparisons with orthogonal relationships (e.g., F0/F180-VF90 in Rot180, Subset 1), the vertical direction of the shears of comparisons including parallel (or vertical) relationships between the picture orientation and the participant orientation (i.e., the picture was oriented upright or upside down with regard to the participant; e.g., F90/F270-VF90 in Rot180, Subset 1) seemed to be less prominent present, regularly resulting in relatively smaller shears. Multiple explanations are possible; for instance, the viewpoint-from-above slant was possibly larger for objects that are oriented horizontally, or perhaps the curvature at the sides of the torso was more subject to slanting. We also observed that from time to time the specific comparisons were somewhat ordered (e.g., Subset 3, Rot90, EC, reference pictures (from top to bottom) F0, F180, F90, F270). Finally, we did not find clear general relationships between the comparisons with an upright or upside down correspondence with the reference frames and the shears (as described in Specific interplay between picture orientation and the environmental and the viewer-centered reference frame section), although comparisons with higher *R*^2^ values for the straight regression (often the comparisons with the correspondences as described earlier) were frequently related to smaller shears between the pictorial reliefs; consider for instance the shears demonstrated by Subset 4, Rot90 Rot180, VF270. To elucidate these latter observations more, further study is required.

As could be expected on basis of the findings from the straight regression, the shear transformation had more impact for the Rot180 comparisons than for the Rot90 and Rot270 comparisons, for all three subsets (Subset 4 is not mentioned since this subset consisted of combinations of differences between picture orientations and differences between picture orientations). The pictorial reliefs of the comparisons within Rot180 thus needed larger shears to transform the pictorial relief of the reference picture toward the pictorial relief of the comparison picture. Also, in spite of the general similar positions of the shears corresponding with the two poses (but less so for E.C. for Subset 3, Rot90 (and thus also Rot270), the shear was generally larger for Pose 60 (dark shear symbols) than Pose 280 (light shear symbols; Table 1); this is consistent with the finding of the higher *R*^2^ values of the straight regression for Pose 280 compared with Pose 60 for both participants.

 Recapitulating the most important findings concerning the nature of the difference (i.e., the shears) between the pictorial reliefs:

 Our results suggest that the viewpoint-from-above assumption plays an obvious, convincing role in explaining the shears in the comparisons between the pictorial reliefs. The viewpoint-from-above assumption describes the human tendency to perceive objects as if they were looked at from above. We proposed that this viewpoint-from-above is (evenly) determined by the environmental and the viewer-centered reference frame. Moreover, the (frontward) slant related to the viewpoint-from-above was considered as the total of the frontward slant of the bottom of the picture, with regard to the environmental reference frame, and the frontward slant of the bottom of the picture, with regard to the participant orientation. In addition, the shear transformation between two pictorial reliefs was found to result from a combination of the (inverse) viewpoint-from-above slant of the reference picture and the viewpoint-from-above slant of the comparison picture.

## General Discussion and Conclusion

In the present study, we aimed to gain more insight in the contribution of the environmental and the viewer-centrred reference frame in (pictorial) shape perception. Therefore, we compared the pictorial reliefs of the depicted object based on various picture and participant orientations. On the basis of the extensive set of comparisons between pictorial reliefs (i.e., 528 in total), we attempted to disentangle the influence of the environmental and the viewer-centered reference frames.

### General Influence of the Environmental and the Viewer-Centered Reference Frame

First, on basis of the *R*^2^ values of the straight regression considered as a measure of the extent of the similarities between the pictorial reliefs, we could deduce that, overall, picture orientation as well as participant orientation influenced the pictorial reliefs. The comparison between Subsets 1 and 2 suggested that the variation in picture orientation (Subset 1) is more distorting than the variation in participant orientation (Subset 2). However, we think it is more interesting to consider the subsets with regard to the relationships between the picture orientation and the environmental and viewer-centered reference frame.

In Subset 2, the picture orientation only varied with respect to the viewer-centered reference frame; in Subset 3, the picture orientation differed with respect to the environmental reference frame. When having disconnected the two reference frames, the environmental (Subset 3) nor the viewer-centered reference frame (Subset 2) seemed to account for large discrepancies between the pictorial reliefs; both subsets showed moderate to strong similarities between the pictorial reliefs. It could be that the decoupling of the environmental (Subset 3) and the viewer-centered reference (Subset 2) frames has a small distorting effect, or that a constant relationship of the picture orientation with either the environmental (Subset 2) or the viewer-centered reference frame (Subset 3) gains dominance over the distorting influence of the viewer-centered or the environmental reference frame, respectively (both explanations are indistinguishable from each other). In contrast, when the picture orientation differed with respect to the environmental as well as to the viewer-centered reference frame (Subsets 1 and 4), the pictorial reliefs showed rather large dissimilarities. Having compared the similarities between the pictorial reliefs of Subsets 2 and 3, with those of Subsets 1 and 4, we came to the conclusion that the interplay between the environmental and the viewer-centered reference frame seemed to be important in explaining the dissimilarities.

The observations described earlier suggest that in everyday life (in which varying object orientations are generally viewed from an upright viewer orientation), the environmental as well as the viewer-centered reference frame play a role in shape perception. If the reference frames are decoupled, and the object orientation is constant with one of both reference frames, the reference frame involving a varying relationship with the object orientation has a rather small influence. However, one must take into account that the stimulus used in the present study is a specific one. Future research could investigate whether the findings discussed here would generalize to other stimuli that do not have a social component or a canonical orientation (e.g., potato-like shapes or peppers). Below, we return to the specificity of the torso in this study in more detail. Nevertheless, on the whole, this study clearly demonstrated that the superiority of the environmental or the viewer-centered reference frame depends on the specific conditions and comparisons. This conclusion is consistent with previous studies (e.g., on the complexity of stimuli: Rock & Heimer, 1957; on the absence of a gravitational reference frame: Friederici & Levelt, 1990; Leone et al., 1995; on the conditions of attention: Rock & Nijhawan, 1989).

Besides the general influence of the environmental and the viewer-centered reference frame described earlier, we observed that some individual comparisons with high similarities between the pictorial reliefs could be characterized by specific correspondences between the picture orientation and the environmental or the viewer-centered reference frame.

### Specific Influence of the Environmental and the Viewer-Centered Reference Frame: The Importance of Verticality

#### Upright picture orientation with regard to the environmental or the viewer-centered reference frame

One of the correspondences related to high similarities between the pictorial reliefs concerned the comparisons with the picture orientations upright with regard to the environmental or the viewer-centered reference frame.

The special relationship of the upright picture orientation with regard to the environmental reference frame appears to be rather evident to explain: The torso is an object with a clear base and a clear canonical orientation (i.e., upright with regard to the environment). More surprisingly, however, the pictorial relief of a picture oriented upright with regard to the viewer-centered reference frame was very similar to the pictorial relief of a picture oriented upright with regard to the environmental reference frame: The viewer-centered reference frame fulfilled the same role as the environmental reference frame.

Rock and Heimer (1957) observed that the phenomenal orientation of the form—specified by the parts that are considered as up and down—could not only be determined by the environmental reference frame but, in specific circumstances (e.g., in the absence of the environmental reference frame), also by the viewer-centered reference frame (for studies reporting on the viewer-centered reference frame taking over in situations of absence of gravity, see Leone et al., 1995; Friederici & Levelt, 1990). In the light of the present findings, we would like to broaden the circumstances mentioned by Rock and Heimer (1957) to include the consistent alignment of the picture orientation with regard to the viewer-centered reference frame. Although in the present study, the participant did not need to compare different shape percepts but only had to perform a direct and visual task, it seems relevant to mention Friedman and Hall (1996) who found that participants with heads tilted by 45° needed less time to discriminate between the 45° tilted same-different pairs compared with the upright participant orientations. More recently, Waszak et al. (2005) observed that participants who had to discriminate between different views of a 3-D object while lying on their side, relied on the viewer-centered reference frame if it was consistent with the visual information (i.e., the participant orientation as well as the visual context was tilted by 90°).

Besides being a mono-oriented object, the torso is also an animate object. Consequently, if a picture is oriented upright with respect to the participant orientation, the participant is in the same orientation as this specific animate object. The studies mentioned earlier used inanimate, unfamiliar, and non-based figures. Another relevant line of (mental rotation) research used animate objects—such as bodies, body parts, and animals—instead of inanimate objects. Generally, it was found that with animate objects, mental rotation did not use an object centered reference frame but a viewer-centered reference frame (Amorim, Isableu, & Jarraya, 2006; Dalecki, Hoffmann, & Bock, 2012; Yu & Zacks, 2010; Zacks, Mires, Tversky, & Hazeltine, 2002). We cannot completely confirm the statement of Rock, Wheeler, and Tudor (1989) that only representation of objects with salient intrinsic axes, such as bodies, could disregard the influence of the environmental reference frame. However, it is clear from our observations that in some specific cases of correspondence of the picture orientation with the viewer-centered reference frame, the influence of the environmental reference frame is indeed minimal. Although bodies—and thus to a certain extent torsi—could be considered as special stimuli, comparable to faces (for a review, see e.g., Minnebusch & Daum, 2009), Lakoff and Johnson (1999) proposed another, more general, possibility: The reference frame as defined by the body of the viewer (i.e., the axes from head to feet, from front to back, and from left to right) could be mapped onto the embodied object which would then have these body axes. (Note that Khetrapal (2010) contrasted this mapping with a first-order projection of relating the bodily axes to an object in order to define it spatially.) A related, metaphorical thought of correspondence between the object and the observer might be found in Lipps’ “Aesthetische Einfühlung” (1913) with the feeling of “empathy” of one’s body when looking at, for instance, a column reaching up.

Up till now, we discussed the similarity between the pictorial reliefs obtained from an upright picture orientation with regard to one or both of the reference frames (i.e., the environmental and the viewer-centered reference frame) and placed this finding in a theoretical framework. However, surprisingly, not only the upright orientation with regard to the viewer or environmental reference frame displayed similar pictorial reliefs; similar pictorial reliefs were also produced when the comparisons included an upside down picture orientation with regard to the viewer or environmental reference frame.

#### Upside down picture orientation with regard to the environmental or the viewer-centered reference frame

Not only the upright picture orientation but also the upside down picture orientation showed a strong perceptual stability with regard to the environmental and the viewer-centered reference frame. This suggests that the axes of elongation or symmetry rather than the canonicality of the torso or the “selection of the parts of a form which are to be its phenomenal top, bottom and sides” (Rock & Heimer, 1957, p. 501), as proposed earlier, are important in providing the stability of a shape percept. (Although, in the present study, we cannot discern between the axes of the image and the torso, we assume that the intrinsic axes of the torso are of main importance.) Clearly, an upright or upside down picture orientation is equivalent to the torso’s axis of elongation (or symmetry) being vertically oriented.

Numerous studies have reported on the importance of the intrinsic axes of elongation or symmetry of—inanimate—objects (e.g., Boutsen & Marendaz, 2001; Humphreys, 1983, 1984; Quinlan & Humphreys, 1993; Shiffrar & Shepard, 1991; Sekuler & Swimmer, 2000; but see also Large, McMullen, & Hamm, 2003). More importantly, Wiser (1981, cited in Leek, 1998, p. 650) reported on the importance of the axis of elongation (and symmetry) in combination with the vertical orientation: Object recognition was faster when the axis of elongation (and symmetry) of the shape was vertically oriented, even when they were presented earlier in an oblique or horizontal orientation. Also, Metzler (1973, cited in Shiffrar & Shepard, 1991, p. 45) found that same different discrimination was more efficient not only when the objects were rotated around one of their own intrinsic axes but also when the axis of rotation was vertical with regard to the environment. Furthermore, objects rotated around the vertical axis were recognized more efficiently, that is, faster (Parsons, 1987; Shiffrar & Shepard, 1991) and more accurate (Bülthoff & Edelman, 1992), than around other axes. Similarly, views of objects obtained by rotation around the vertical, with respect to gravitation (in upright or tilted positions but without visual context) or the visual context (when aligned with the viewer), were recognized faster than views obtained by rotation around the horizontal axis (Waszak et al., 2005). In addition, imagined self-rotation was reported to be easier around the vertical axis than around the horizontal axis (Creem, Wraga, & Proffitt, 2001).

Besides the (mental) rotation and object recognition literature (which is quite different from the direct viewing mode investigated here), the visual perception literature has discussed the special status of verticality at great length (e.g., Mach, 1886/1959). Mirror reflection around the vertical symmetry is also omnipresent in nature, considered to fulfil a special role in, for instance, human facial attractiveness (Perrett et al., 1999) or sexual selection of various species (Møller & Thornhill, 1998). In addition, an extensive set of studies has demonstrated the salience of vertical symmetry in visual perception (see for detection studies using dot patterns, e.g., Corballis & Roldan, 1975; Wagemans, van Gool, & d’Ydewalle, 1992, Gabor patch arrays, e.g., Machilsen, Pauwels, & Wagemans, 2009, geometrical figures, e.g., Palmer & Hemenway, 1978, or biological shapes, e.g., Evans, Wenderoth, & Cheng, 2000; Wilson & Wilkinson, 2002). Moreover, in Cornelis et al. (2003, 2009), the pictorial reliefs based on pictures that differed from each other by a mirror reflection around the vertical axis were found to be very similar, once again indicating the special status of vertical symmetry.

Interestingly, from a completely different angle of study than the ones we just referred to, in the present study, we also found evidence for the special status of the verticality of the depicted object, that is, the torso, and consequently of its intrinsic axis of elongation. Moreover, the vertical orientation of the object was not only special with respect to the environmental but also to the viewer-centered reference frame; both reference frames exerted the same influence on the shape percept of the depicted object. It is most likely that the salience of the verticality with regard to both the environmental and the viewer-centered reference frame is related to our interaction with our environment. After all, the environmental reference frame usually coincides with the viewer-centered reference frame. Shiffrar and Shepard (1991, p. 44) consider that “the influential role of the vertical dimension may ultimately arise from the terrestrial gravitational field, which (as emphasized by Shepard, 1982, 1984) has remained an invariant of our physical environment throughout biological evolution.” Possibly, humans are so used to orient themselves upright with respect to the world and the objects around it, that this vertical orientation with regard to the viewer-centered reference frame becomes similarly important as the vertical orientation with regard to the environmental reference frame (Kant, 1781/1881, mentioned in Shepard & Hurwitz, 1984, p. 161).

In this study, the importance of verticality was deduced from the extent of the (dis)similarity between two pictorial reliefs. We also examined the nature of the (dis)similarity between the pictorial reliefs, which we discuss in the next section.

#### Interpretation of the differences between the pictorial reliefs: The mental viewpoint as the viewpoint-from-above

Closer examination of the differences between the pictorial reliefs within the various comparisons revealed that not only the depth dimension but also the image plane played a role. Shears between the pictorial reliefs, generally explaining the differences between the pictorial reliefs best, could be considered as a combination of the slants originating from the reference and the comparison picture. It seemed that the bottom of all pictures, whatever their orientation, was slanted to the front (or the top of the picture was slanted to the back). The frontward slant of the bottom of the pictorial relief supports the assumption that humans have a tendency to perceive the object as one was viewing it from above. Interestingly, the viewpoint-from-above was determined, independent of the canonical or more natural orientation of the object, with respect to the environment as well as with respect to the participant. Furthermore, the environmental and the viewer-centered reference frame functioned in the same way to define this viewpoint; this was especially obvious from Subset 1.

Although Troje and McAdam (2010) rightly state that the viewing-from-above bias has not yet been given a lot of attention in the research literature, some studies are noteworthy to mention. Mostly, unlike our study, the viewpoint-from-above is mentioned in relation to ambiguous stimuli. The viewpoint-from-above prior would, for instance, be responsible for the bias of interpreting a Necker cube mostly with the top surface as the top face (e.g., Martelli, Kubovy, & Claessens, 1998; under conditions of presentation with no context: Sundareswara & Schrater, 2008). The spinning dancer is more likely to rotate clock-wise, corresponding with a view-from-above (Troje & McAdam, 2010). In addition, Mamassian and Landy (1998) found evidence for the viewpoint-from-above prior in the perception of line drawings that could be interpreted as surface patches. Although we did not present ambiguous stimuli,^16^ the torso was perceived differently when presented in different orientations. We regarded the differences between the pictorial reliefs as related to the viewpoint-from-above, which was determined by the environmental and the viewer-centered reference frame. As far as we know, the viewpoint-from-above prior was not discussed before in combination with variation in participant orientations, meaning that the viewpoint-from-above was only demonstrated in the case of the environment coinciding with the participants’ orientation (comparable to the Subset 1, VF0 participant orientation in our study). We consider the disentanglement of the environmental and the viewer-centered reference frame concerning the viewpoint-from-above an interesting addition to the literature.

Following Koenderink et al. (2001), Cornelis et al. (2003, 2009) suggested that the shear possibly indicated a relocation of the “mental eye” so that the “mental viewing direction” would be orthogonal to the tangent plane of the surface. In Koenderink et al. (2001), it was suggested that the “mental viewpoint” corresponded with a canonical view of the object (i.e., “The Turtle” of Brancusi), that is, above its symmetry axis. However, in the present study, the shears were largely defined by the viewpoint-from-above slants, which, in turn, were determined by the top-bottom direction of the picture. Mostly, the viewpoint-from-above was not influenced by the specific orientation of the depicted object; it was the top of the picture (or the depicted object), whatever its orientation, that slanted to the back. The semantic content, although some variation in the shears might be ascribed to it, was of minimal importance. The fact that the general trend in the shears depended mostly on the transformation is understandable, considered from the viewpoint-from-above interpretation.

Moreover, as said before, the viewpoint-from-above was determined by the environmental as well as the viewer-centered reference frame. Liu and Todd (2004) speculated that it is more common to observe surfaces from above rather than below and that the perception of ambiguous images occurs in such a way so that the surface depth increases with the height in the image plane. Since the environmental and the viewer-centered reference frame are usually aligned in everyday circumstances, the viewpoint-from-above might be strongly connected to both reference frames (see also earlier). If the viewpoint-from-above prior is indeed related to assigning more depth the higher one gets in the image plane, it seems that the visual system applies this rule systematically, and independent of the orientation of the (familiar) depicted object.

Earlier, we were wondering if the depicted object, that is, the torso, we have used, was giving rise to possible artifacts. From the view of the shears, we feel rather fortunate to have chosen this specific object: Even in the case of a (social) meaningful object with a particular natural, canonical, orientation, and with a clearly visible base, the shears were mostly dependent on the transformations, with the viewpoints-from-above (almost) not related to the content of the picture.

Nevertheless, it would be interesting to use other (meaningless as well as meaningful) objects with varying degrees of complexity of surface structure. Although it is clear from this study (and previous work by Cornelis et al., 2003) that piecewise differences almost completely disappear after affine transformation,^17^ these piecewise differences might be worthwhile to examine further. Furthermore, it could be interesting to apply the gauge figure method (or another method by which the 3-D shape percept can be externalized) in combination with the recognition paradigms often used to investigate the perceptual differences between different presentations (such as orientations) of, for instance, faces.

In the context of the relocation of the “mental viewpoint,” we previously (Cornelis et al., 2009; Koenderink et al., 2001) referred to the *beholder’s share*. In the present study, we considered the shears between the pictorial reliefs to be closely related to a prior, that is, the viewpoint-from-above. In addition, despite interindividual differences, the participants solved the picture ambiguities in much the same way. Therefore, one could conclude that, the beholder’s share in solving picture ambiguities in the 3-D shape perception of pictured objects, appears to be largely driven by general a priori principles. Nevertheless, further research should be encouraged to generalize the present findings to larger samples.

## Conclusion

From the study of the comparisons between the pictorial reliefs obtained from different participant orientations and picture orientations, we came to the following conclusions:

 From investigating the extent of the (dis)similarities, we found that the verticality of the depicted object (i.e., a torso) with regard to the environmental or the viewer-centered reference frame was related to high similarities between the pictorial reliefs. In those cases, the environmental and the viewer-centered reference frame fulfilled the same function. Interestingly, not only the canonical upright picture orientation but also the upside down picture orientation corresponded with stability between the pictorial reliefs.

 From investigating the nature of the (dis)similarities, we interpreted the shears as concatenations of slants generated from the viewpoint-from-above. Following this viewpoint-from-above interpretation, we found that the semantic content of the picture (and thus the specific orientation of the depicted object) was of minimal importance in determining the viewpoint-from-above. The backward slant of the top of the picture generated by the viewpoint-from-above was predominantly defined by the environmental as well as the viewer-centered reference frame.

## Supplementary Material

Supplementary material

## Supplementary Material

Supplementary material

## Supplementary Material

Supplementary material

## Supplementary Material

Supplementary material

## Supplementary material

The online appendices are available at http://IPE/sagepub.com/supplemental

## Notes

1. We chose to include both the left-hand side (VF90) and the right-hand side (VF270) participant orientations as well as both the 90° (F90) and the 270° (F270) rotated versions of the original (upright) photograph (F0; with V for viewer; F for fiducial in the abbreviations). Besides attempting to avoid possible artifacts when including only one of the side orientations (of the participant or the picture), it gave us the opportunity to consider more comparisons and provide more strength to our findings regarding the effect of a rotation of 180° (Rot180) of the picture and the participant (Note: the upside down participant orientation was not included, since we considered this too unnatural and burdensome. In contrast, the comparison between the upright (F0) picture orientation and the upside down (F180) picture orientation was included). In addition, we expected the inclusion of both side orientations to enable us to shed some light onto the effect of the lighting direction on the shape percept of the depicted object (see Specific interplay between picture orientation and the environmental and viewer-centered reference frames section).

2. Since we included all possible comparisons and gathered pictorial reliefs from all possible combinations of picture orientations and participant orientations (i.e., 3 × 4), one would expect 144 (i.e., 12 × 12) comparisons. However, since we did not compare the pictorial relief with itself (see the gray boxes in Figure 6 and the red font in Table 2 in the Supplement), we arrived at 132 comparisons in total. For each of the 12 pictorial reliefs (indicated as reference at the left of Table 2 in the Supplement), there are 11 comparison pictorial reliefs.

3. Since the straight regression included the intercept, *R*^2^ is equal to the square of the correlation coefficient (*r*) between the depth lists of the comparison and the reference pictorial relief.

4. Since the straight regression describes the straightforward relationship between the depth lists (and consequently considers depth scaling), we have chosen the *R*^2^ of the straight regression as a summarizing measure of similarity between the pictorial reliefs. When we mention that the pictorial reliefs within a comparison are similar, on the basis of a high *R*^2^ value of the straight regression, we thus mean that the depth list of the comparison pictorial relief can be well-predicted by the depth list of the reference pictorial relief. This notion of similarity does not reveal the precise nature of the relationship (which is defined by the weight ‘*d*’ in z_comp_ = a + dz_ref_); we only make inferences about the strength of the relationship between the depth lists. In other words, our notion of similarity does not exclude possible depth scaling effects; the nature of the relationship of highly similar pictorial reliefs can be specified by a depth flattening, a depth deepening or a depth identity.

5. All the *R*^2^ values of the straight regression occurred twice. For every comparison, there was an inverse comparison that resulted in the same *R*^2^ value for the straight regression. For instance, the comparison between the pictorial reliefs obtained from the F0 picture and the F90 picture (Rot90) demonstrated the same *R*^2^ value for the straight regression as the comparison between the pictorial reliefs from the F90 picture and the F0 picture (Rot270). As one can notice in Figure 5, the distribution of the comparisons across the various (ODpict and ODpart) categories was moderately balanced: The ODpict − ODpart combinations involved either 32 or 48 comparisons. However, eliminating the inverse comparisons would lead to a different, and seriously unbalanced, distribution. When having eliminated the inverse comparisons, ODpict and ODpart would possibly contain the following categories (with the number of comparisons, per pose, per participant, within brackets): ODpict: Rot0 (12): Rot90 (8), Rot180 (4); Rot90 (36): Rot0 (12), Rot90 (8), Rot180 (8) Rot270 (8); Rot180 (18): Rot0 (6), Rot 180 (4), Rot90 (8); ODpart: Rot0 (18): Rot90 (12), Rot180 (24); Rot90 (24): Rot0 (8), Rot90 (8), Rot180 (8), Rot270 (8); Rot180 (16), Rot0 (4), Rot90 (8), Rot180 (4); Rot270 (8): Rot90 (8). As one can notice in the list earlier, the Rot270 category within ODpart, for instance, would only contain the Rot90 (ODpict) comparisons. To avoid such small numbers of data within particular categories of comparisons, we have chosen to include the inverse comparisons and thus the duplicate *R*^2^ values in the analysis.

6. Part of the results of Subset 1 were previously reported in Cornelis et al. (2009), in which we investigated the effect of planar rotations of a picture on the shape percept of the depicted object, with the participant sitting straight up (VF0). In the present study, we extended the comparisons by including two other orientations of the participant: lying on the right-hand side (VF90) and lying on the left-hand side (VF270). For the sake of completeness, the comparisons between picture orientations with the participant oriented upright (VF0) are included here as well.

7. In addition to the multilevel analysis on the complete data set, we conducted a multilevel analysis on the reduced data set. In contrast to the previous analysis with ODpict and ODpart as independent variables, elimination of the duplicate *R*^2^ values was possible in this case because the eliminated *R*^2^ values were evenly distributed over the subsets; for each subset, the number of *R*^2^ values was reduced to half. However, analysis on this reduced data set led to approximately the same results: a significant main effect for Subset, *F*(3, 259) = 13.320, *p* < .0001) with Subset 1 significantly differing from Subset 2 (*p* = .012) and Subset 3 (*p* < .0001) as well as Subset 4 significantly differing from Subset 2 (*p* = .002) and Subset 3 (*p* < .0001).

8. Please note that not all comparisons specified in this section, and in the following sections, show evenly distinct *R*^2^ values. Because it is fairly easy to deduce from the bar charts, we have chosen not to explicitly mention the comparisons with less consistent magnitudes of *R*^2^ values.

9. These comparisons were responsible for the higher mean *R*^2^ value of Subset 4 for Rot180 observed in Figure 7.

10. Please note that we have explicitly chosen not to control for the position of the light source, as this would inherently encompass making different photographs. Changing the position of the light source between photographs would result in different shading patters onto the depicted object (see for the effect of different lighting conditions, Koenderink et al., 1996; Todd et al., 1996). This adjustment would not fit the focus of the present study, that is, investigating the effect of orientation of the (same) picture with regard to the environmental and the viewer-centered reference frame on the shape percept of the depicted object.

11. We would like to stress that the assumption of perceptual equivalence between F90 and F270, and between VF90 and VF270 does not entail perceptual similarity: both F90 and F270 and VF90 and VF270 differ by 180°, and as mentioned earlier, the Rot180 comparisons were related to considerable differences between pictorial reliefs.

12. Please note that, although the torso is lit from left above (see the original photograph F0), we only mention the lighting from above. Obviously, the lighting from above is inextricably linked to the lighting from the left (see also Table 4 in the Supplement).

13. Please note that Rot270 contains the inverse comparisons of Rot90. One might assume that the shear symbols of Rot90 would be rotated by 180° to obtain the shear symbols of Rot270. However, as mentioned earlier, the *x* and *y* axis of the polar plots refer to the horizontal and the vertical dimensions of the image plane, whatever the reference orientation of the picture or the participant may be. Consider, for instance, comparison Rot90, F0/F90-VF0 and the inverse comparison Rot270, F90/F0-VF0. The shear symbol related to the Rot90 comparison is positioned in the left lower quadrant of the plot: The right upper corner of the pictorial relief of the reference picture F0 has to be slanted to the front in order to become similar to the pictorial relief of the comparison picture F90. Evidently, the Rot270 comparison exhibited the opposite slant, that is, the left upper corner of the pictorial relief of the reference picture F90 has to be slanted to the front to change into the pictorial relief of the comparison picture F0. The shear symbol related to this opposite slant is situated in the right lower corner because the reference picture is F90.

14. For the sake of consistency with the description of the shears, we refer to the frontward slants of the bottom instead of the backward slants of the top.

15. The shears we calculated expressed the transformation of the reference pictorial relief toward the comparison pictorial relief. Therefore, the viewpoint-from-above slant related to the reference picture had to be inverted before adding up to the slant of the comparison pictorial relief. Contradicting slants thus refer to highly similar slants of the reference pictorial relief and the comparison pictorial relief.

16. In the sense that we presented a meaningful object. Of course, as mentioned, a picture inherently involves picture ambiguities.

17. For the sake of completeness, we would like to add that, previously (Cornelis et al., 2003; Koenderink & van Doorn, 2003), it was found that the residuals obtained after applying the affine transformation were related to specific surface regions.
