# Supplementary material for: Reference Frames and 3-D Shape Perception of Pictured Objects: On Verticality and Viewpoint-From-Above
Source: Iperception. 2016 Jun 29;7(3):2041669516637286. doi: 10.1177/2041669516637286 (PMC4934666; doi:10.1177/2041669516637286)
Supplement: Supplementary material [file i0770_FN_Suppl_Table_1.pdf]

Table 1. Overview of some concepts / notations.

concept / notation	explanation
Pose 60<br>Pose 280	frontal pose of the torso<br>dorsal pose of the torso
F0<br>F90<br>F180<br>F270	picture orientations:<br>original photograph<br>original photograph rotated by 90 degrees<br>original photograph rotated by 180 degrees<br>original photograph rotated by 270 degrees<br>(with F for fiducial)
VF0<br>VF90<br>VF270	participant orientations:<br>sitting straight up<br>lying on the right-hand side<br>lying on the left-hand side<br>(with F for fiducial, V for viewer)<br>Please note we did not include the upside down orientation of the participant (VF180).
AD and EC	participants
gauge figure	consisted of the outline of an ellipse with a line segment sticking out from the centre of the ellipse. The task consisted of adjusting this gauge figure, superpositioned onto the depicted torso, so that it looked as if fitting in the scene.
slant and tilt	attitude parameters as indicated by the gauge figure settings, gathered for every condition (i.e. combination of picture orientation x participant orientation). On basis of the slant and tilt values collected over the entire area of the depicted object in one session, depth gradients were calculated at each probe's position.
pictorial relief	externalized 3-D percept of the depicted object
regression analyses<br>straight regression	The simple (straight) regression analyses related the depth values of the comparison picture to the depth values of the reference picture ($z_{comp} = a + dz_{ref}$, with z representing the depth values). For every comparison there was an inverse comparison that resulted in the same R^2 value for the straight regression (the inverse comparisons are represented between brackets in the figures). In the text, we only mention one of the two comparisons related to that same R^2 value, i.e. the first comparison with the lowest difference between the picture orientations followed by the lowest difference between the participant orientations. For instance, we mention the comparisons of the Rot90 category but not the inverse comparisons belonging to the Rot270 category.
affine regression	The multiple (affine) regression analysis ($z_{comp} = a + bx + cy + dz_{ref}$, with z representing the depth values; with the image coordinates x and y, measured in screen pixels, referring to the positions on the image plane on which the depth values were calculated) took into account not only the depth dimension, but also the x- and y-dimension of the image plane. This regression analysis was considered as an affine transformation because of its geometrical properties: invariance of parallelism, collinearities and ratios. In contrast with the straight regression, the affine regression is not entirely reversible; therefore, the inverse comparisons did not result in duplicate R^2 values.
ODpict<br>ODpart	difference between picture orientations<br>difference between participant orientations
Rot0<br>Rot90<br>Rot180<br>Rot270	rotation categories, referring to ODpict and / or ODpart, indicating the degree of the difference between orientations.

Subset 1<br>Subset 2<br>Subset 3<br><br>Subset 4	The comparisons between pictorial reliefs were divided in four subsets:<br>comparisons with only the picture orientation varied<br>comparisons with only the participant orientation varied<br>comparisons with the picture orientation varied to the same extent as the participant orientation<br>comparisons with both the picture orientation and the participant orientation varied independently of each other
specific comparisons	are noted by an abbreviated notation, with “ /” referring to the comparison between reference and comparison and with “ - ” referring to the combination of picture orientation and participant orientation in either the reference or the comparison or both.
for instance:<br>F0/F90-VF0	indicates the comparison (of Subset 1) between the pictorial reliefs based on pictures differing by 90 degrees, with reference picture F0 and comparison picture F90, and participant orientation VF90.
F0/VF0-VF90	indicates the comparison (of Subset 2) between the pictorial reliefs based on the same picture orientation that was looked at from two different participant orientations, VF0 and VF90. The reference picture is thus the same as the comparison picture, i.e.F0. The reference participant orientation is VF0 and the comparison participant orientation VF90.
F0-VF0/F90-VF90	indicates the comparison (of Subset 3) between the pictorial reliefs with reference picture orientation F0 and reference participant orientation VF0; the comparison picture orientation is VF90. The difference between picture orientations is the same as the difference between participant orientations.
F0-VF90/F90-VF270	indicates the comparison (of Subset 4) between the pictorial reliefs with reference picture orientation F0 and reference participant orientation VF90; the comparison picture orientation is F90 and the comparison participant orientation is VF270. The orientations between pictures thus differ 90 degrees; the orientations between participants differ 180 degrees.
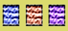	indicate the R^2 values of the straight regression of the comparisons with a correspondence between the picture orientations and the viewer centred reference frame on the one hand and the environmental reference frame on the other hand, with one of the picture orientations upright (F0) or upside down (F180) according to the environmental reference frame (Figure 7 (a), (c), (d)).<br>In the text, this texture is denoted by ‘a fine, horizontal texture’.
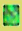	indicates the R^2 values of the straight regression of the comparisons with a correspondence between the picture orientation and the environmental reference frame, with the picture orientation F0 or F180 (Figure 7 (b)).<br>In the text, this texture is denoted by ‘a clumped grain texture’.
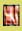	indicates the R^2 values of the straight regression of the comparisons with a correspondence between the picture orientations and the viewer centred reference frame, with the picture orientation upright or upside down with regard to the participant orientation (Figure 7 (c)).<br>In the text, this texture is denoted by ‘a rough random texture’.
shear	was calculated from the weights *b* and *c* of the image coordinates *x* and *y* in the affine regression. The arctangent of the ratio of the weights *c* and *b* determined the direction of the shear; a measure for the magnitude of the shear was limited between 0 and 1 by calculating $\sin\{\arctan[\sqrt{(b^2 + c^2)}]\}$.
attitude change	is used in the context of the behaviour of the shear
slant	is used in the context of the viewpoint-from-above (different from the previously used concept of ‘slant’ (see above))
