# Supplementary material for: Reference Frames and 3-D Shape Perception of Pictured Objects: On Verticality and Viewpoint-From-Above
Source: Iperception. 2016 Jun 29;7(3):2041669516637286. doi: 10.1177/2041669516637286 (PMC4934666; doi:10.1177/2041669516637286)
Supplement: Supplementary material [file i0770_FN_Suppl_Table_2.pdf]

Reference		Comparison					
Pict	Part	Pict	Part	Pict	Part	Pict	Part
F0	VF0	F0	VF0	F0	VF90	F0	VF270
F0	VF0	F90	VF0 *	F90	VF90	F90	VF270
F0	VF0	F180	VF0	F180	VF90	F180	VF270
F0	VF0	F270	VF0	F270	VF90	F270	VF270
F90	VF0	F0	VF0 **	F0	VF90	F0	VF270
F90	VF0	F90	VF0	F90	VF90	F90	VF270
F90	VF0	F180	VF0	F180	VF90	F180	VF270
F90	VF0	F270	VF0	F270	VF90	F270	VF270
F180	VF0	F0	VF0	F0	VF90	F0	VF270
F180	VF0	F90	VF0	F90	VF90	F90	VF270
F180	VF0	F180	VF0	F180	VF90	F180	VF270
F180	VF0	F270	VF0	F270	VF90	F270	VF270
F270	VF0	F0	VF0	F0	VF90	F0	VF270
F270	VF0	F90	VF0	F90	VF90	F90	VF270
F270	VF0	F180	VF0	F180	VF90	F180	VF270
F270	VF0	F270	VF0	F270	VF90	F270	VF270
F0	VF90	F0	VF90	F0	VF270	F0	VF0
F0	VF90	F90	VF90	F90	VF270	F90	VF0
F0	VF90	F180	VF90	F180	VF270	F180	VF0
F0	VF90	F270	VF90	F270	VF270	F270	VF0
F90	VF90	F0	VF90	F0	VF270	F0	VF0
F90	VF90	F90	VF90	F90	VF270	F90	VF0
F90	VF90	F180	VF90	F180	VF270	F180	VF0
F90	VF90	F270	VF90	F270	VF270	F270	VF0
F180	VF90	F0	VF90	F0	VF270	F0	VF0
F180	VF90	F90	VF90	F90	VF270	F90	VF0
F180	VF90	F180	VF90	F180	VF270	F180	VF0
F180	VF90	F270	VF90	F270	VF270	F270	VF0
F270	VF90	F0	VF90	F0	VF270	F0	VF0
F270	VF90	F90	VF90	F90	VF270	F90	VF0
F270	VF90	F180	VF90	F180	VF270	F180	VF0
F270	VF90	F270	VF90	F270	VF270	F270	VF0
F0	VF270	F0	VF270	F0	VF0	F0	VF90
F0	VF270	F90	VF270	F90	VF0	F90	VF90
F0	VF270	F180	VF270	F180	VF0	F180	VF90
F0	VF270	F270	VF270	F270	VF0	F270	VF90
F90	VF270	F0	VF270	F0	VF0	F0	VF90
F90	VF270	F90	VF270	F90	VF0	F90	VF90
F90	VF270	F180	VF270	F180	VF0	F180	VF90
F90	VF270	F270	VF270	F270	VF0	F270	VF90
F180	VF270	F0	VF270	F0	VF0	F0	VF90
F180	VF270	F90	VF270	F90	VF0	F90	VF90
F180	VF270	F180	VF270	F180	VF0	F180	VF90
F180	VF270	F270	VF270	F270	VF0	F270	VF90
F270	VF270	F0	VF270	F0	VF0	F0	VF90
F270	VF270	F90	VF270	F90	VF0	F90	VF90
F270	VF270	F180	VF270	F180	VF0	F180	VF90
F270	VF270	F270	VF270	F270	VF0	F270	VF90
