# Supplementary material for: Reference Frames and 3-D Shape Perception of Pictured Objects: On Verticality and Viewpoint-From-Above
Source: Iperception. 2016 Jun 29;7(3):2041669516637286. doi: 10.1177/2041669516637286 (PMC4934666; doi:10.1177/2041669516637286)
Supplement: Supplementary material [file i0770_FN_Suppl_Table_3.pdf]

Table 1. Pairs of comparisons including different lighting directions with regard to the environmental and / or the viewer centred reference frame.

		comparison	1st part comparison	light source with respect to E	light source with respect to V	2nd part comparison	light source with respect to E	light source with respect to V	R^2 AD 60	R^2 AD 280	R^2 EC 60	R^2 EC 280
**Subset 1**												
1	E V	F0/F90-VF0	F0-VF0	Top	Top	F90-VF0	Right	Right	.052	.520	.537	.467
		F0/F270-VF0	F0-VF0	Top	Top	F270-VF0	Left	Left	.416	.871	.429	.099
									.364	*.351*	*.108*	*.369*
2	E V	F0/F90-VF90	F0-VF90	Top	Left	F90-VF90	Right	Top	.859	.882	.820	.719
		F0/F270-VF270	F0-VF270	Top	Right	F270-VF270	Left	Top	.883	.981	.616	.810
									.024	*.099*	*.204*	*.091*
3	E V	F0/F90-VF270	F0-VF270	Top	Right	F90-VF270	Right	Bottom	.003	.310	.001	.091
		F0/F270-VF90	F0-VF90	Top	Left	F270-VF90	Left	Bottom	.064	.116	.003	.239
									.061	*.194*	*.002*	*.147*
4	E V	F90/F180-VF0	F90-VF0	Right	Right	F180-VF0	Bottom	Bottom	.646	.745	.006	.011
		F270/F180-VF0	F270-VF0	Left	Left	F180-VF0	Bottom	Bottom	.098	.486	.063	.229
									.548	*.259*	*.057*	*.218*
5	E V	F90/F180-VF90	F90-VF90	Right	Top	F180-VF90	Bottom	Right	.186	.272	.189	.180
		F270/F180-VF270	F270-VF270	Left	Top	F180-VF270	Bottom	Left	.332	.591	.066	.075
									.147	*.319*	*.123*	*.105*
6	E V	F90/F180-VF270	F90-VF270	Right	Bottom	F180-VF270	Bottom	Left	.305	.617	.496	.544
		F270/F180-VF90	F270-VF90	Left	Bottom	F180-VF90	Bottom	Right	.783	.547	.636	.758
									.478	*.070*	*.140*	*.214*
7	V	F0/F180-VF90	F0-VF90	Top	Left	F180-VF90	Bottom	Right	.253	.062	.124	.259
		F0/F180-VF270	F0-VF270	Top	Right	F180-VF270	Bottom	Left	.376	.612	.029	.260
									.123	*.551*	*.094*	*.001*
8	E	F90/F270-VF90	F90-VF90	Right	Top	F270-VF90	Left	Bottom	.102	.240	.102	.385
		F270/F90-VF270	F270-VF270	Left	Top	F90-VF270	Right	Bottom	.051	.279	.067	.109
									.051	*.038*	*.035*	*.275*
**Subset 2**												
9	V	F0-VF0/VF90	F0-VF0	Top	Top	F0-VF90	Top	Left	.862	.930	.486	.625
		F0-VF0/VF270	F0-VF0	Top	Top	F0-VF270	Top	Right	.871	.976	.659	.671
									.009	*.046*	*.173*	*.046*
10	E V	F90-VF0/VF90	F90-VF0	Right	Right	F90-VF90	Right	Top	.016	.593	.383	.747
		F270-VF0/VF270	F270-VF0	Left	Left	F270-VF270	Left	Top	.424	.823	.476	.065
									.408	*.230*	*.093*	*.682*
11	E V	F180-VF0/VF90	F180-VF0	Bottom	Bottom	F180-VF90	Bottom	Right	.926	.746	.927	.942
		F180-VF0/VF270	F180-VF0	Bottom	Bottom	F180-VF270	Bottom	Left	.323	.668	.469	.741
									.603	*.078*	*.458*	*.200*
12	V	F270-VF0/VF90	F270-VF0	Left	Left	F270-VF90	Left	Bottom	.229	.449	.439	.429
		F90-VF0/VF270	F90-VF0	Right	Right	F90-VF270	Right	Bottom	.666	.727	.132	.021
									.437	*.278*	*.307*	*.408*
14	E	F90-VF90/VF270	F90-VF90	Right	Top	F90-VF270	Right	Bottom	.119	.299	.088	.073
		F270-VF270/VF90	F270-VF270	Left	Top	F270-VF90	Left	Bottom	.027	.227	.000	.213
									.091	*.072*	*.087*	*.140*
**Subset 3**												
15	E	F0-VF0/F90-VF90	F0-VF0	Top	Top	F90-VF90	Right	Top	.905	.977	.352	.632
		F0-VF0/F270-VF270	F0-VF0	Top	Top	F270-VF270	Left	Top	.868	.975	.389	.812
									.037	*.002*	*.037*	*.180*
16	E	F180-VF0/F270-VF90	F180-VF0	Bottom	Bottom	F270-VF90	Left	Bottom	.915	.849	.745	.856
		F180-VF0/F90-VF270	F180-VF0	Bottom	Bottom	F90-VF270	Right	Bottom	.979	.871	.862	.892
									.064	*.022*	*.117*	*.036*
17	V	F0-VF90/F180-VF270	F0-VF90	Top	Left	F180-VF270	Bottom	Left	.277	.493	.055	.114
		F0-VF270/F180-VF90	F0-VF270	Top	Right	F180-VF90	Bottom	Right	.023	.270	.014	.196
									.254	*.223*	*.041*	*.082*

Note: The difference in the position of the light source between comparisons is defined with respect to the environmental (E) and / or the viewer centred (V) reference frame. Although the torso was lit from left above (see the original photogaph F0), we only refer to the top lighting. Obviously, one can deduce the orientation of the lighting from the left from the orientation of the lighting from the top, with'Top' equivalent to the lighting from the left at the left with respect to E or V; 'Bottom' to the lighting from the left at the right; 'Left' to lighting from the left at the bottom and 'Right' to lighting from the left at the top. For each pair of comparisons, the R^2 value in italics refers to the absolute value of the difference between the R^2 values presented above. The highest R^2 value within each pair of comparisons is indicated by colour. Please note that this table only represents the pairs of comparisons based on interchanging both F90 by F270 and VF90 by VF270.
