# Supplementary material for: Reference Frames and 3-D Shape Perception of Pictured Objects: On Verticality and Viewpoint-From-Above
Source: Iperception. 2016 Jun 29;7(3):2041669516637286. doi: 10.1177/2041669516637286 (PMC4934666; doi:10.1177/2041669516637286)
Supplement: Supplementary material [file i0770_FN_Suppl_Table_4.pdf]

Table 4. Overview of similarities between lighting directions.

	AD		EC	
	Top	Bottom	Top	Bottom
Right	from left (4, 11, 12, 16)	from above (4, 11, 12, 16)	from above (1, 9, 10)<br>from left (11, 16)	from above (11, 16)<br>from left (4, 12)
Left	from above (1, 10)	from left (1, 10)	from above (1, 9, 10)	from above (4, 12)

Note: ‘From above’ and ‘from left’ refer to the lighting from left above of the torso in the original photograph F0. Obviously, the left side lighting is inextricably related to the top lighting in the original photograph F0. ‘Right’ and ‘Left’, and ‘Top’ and ‘Bottom’, indicate the orientation of the lighting. The numbers in superscript refer to the pairs of comparisons from which the similarities are deduced. All similarities are to be considered in relation to a reference, i.e., ‘from left (4, 11, 12, 16)’ in the left corner of the left table (AD) indicates a higher similarity between the right and the top orientation of the left lighting compared to the right and the bottom orientation (see Table 3 in the Supplement).
